# High-performance large-area quasi-2D perovskite light-emitting diodes

**DOI:** 10.1038/s41467-021-22529-x

**Published:** 2021-04-13

**Authors:** Changjiu Sun, Yuanzhi Jiang, Minghuan Cui, Lu Qiao, Junli Wei, Yanmin Huang, Li Zhang, Tingwei He, Saisai Li, Hsien-Yi Hsu, Chaochao Qin, Run Long, Mingjian Yuan

**Affiliations:** 1 Key Laboratory of Advanced Energy Materials Chemistry (Ministry of Education), Renewable Energy Conversion and Storage Center (RECAST), College of Chemistry, Nankai University, Tianjin, P. R. China; 2 Henan Key Laboratory of Infrared Materials and Spectrum Measures and Applications, School of Physics, Henan Normal University, Xinxiang, P. R. China; 3 Key Laboratory of Theoretical & Computational Photochemistry of Ministry of Education, College of Chemistry, Beijing Normal University, Beijing, P. R. China; 4 School of Energy and Environment & Department of Materials Science and Engineering, City University of Hong Kong, Hong Kong, P. R. China

**Keywords:** Materials for devices, Lasers, LEDs and light sources

## Abstract

Serious performance decline arose for perovskite light-emitting diodes (PeLEDs) once the active area was enlarged. Here we investigate the failure mechanism of the widespread active film fabrication method; and ascribe severe phase-segregation to be the reason. We thereby introduce L-Norvaline to construct a COO^−^-coordinated intermediate phase with low formation enthalpy. The new intermediate phase changes the crystallization pathway, thereby suppressing the phase-segregation. Accordingly, high-quality large-area quasi-2D films with desirable properties are obtained. Based on this, we further rationally adjusted films’ recombination kinetics. We reported a series of highly-efficient green quasi-2D PeLEDs with active areas of 9.0 cm^2^. The peak EQE of 16.4% is achieved in *<n* > = 3, represent the most efficient large-area PeLEDs yet. Meanwhile, high brightness device with luminance up to 9.1 × 10^4^ cd m^−2^ has achieved in *<n*> = 10 film.

## Abstract (web summary)

Performance of perovskite LED tends to decline as the active area increases, thus understanding the failure mechanism is paramount to surmount this limitation. Here, the authors report severe phase-segregation to be the cause, and introduce L-Norvaline to overcome it, as the result, highly-efficient 9.0 cm^2^ green PeLED is realised.

## Introduction

Metal halide perovskite materials have been considered as one of the most promising candidates for next-generation light-emitting diodes (LEDs) application, owing to their superior optoelectronic properties^1–8^. The external quantum efficiency (EQE) of perovskite LEDs (PeLEDs) has already exceeded 20% after several years of development^9–12^. Accordingly, PeLED-based lighting and display innovation could function as an excellent complement to the current technologies, thanks to their high performance. However, the very efficient devices are still restricted to small device where the active areas were currently limited to a few square millimeters. Serious performance decline was always observed when attempted to fabricate large-area PeLEDs. Although novel technologies such as blade-coating have provided a new idea for large-area perovskite LED fabrication, the device performance derived from these methods is still far inferior to that of the spin-coated devices^13^. The small area significantly impedes their commercialization, particularly hindering their application in display and solid-state lighting which require large emitting-area. The problem urgently required a remedy.

Quasi-2D perovskite films with self-assembled multiple-quantum-well structure represent an important category of perovskites and have achieved great success in small-area PeLEDs due to their outstanding optical properties^14–19^. Highly emissive quasi-2D films can be obtained via simple spin-coating by carefully regulating their crystallization kinetics. More importantly, the fabrication does not require any additional purification or ligand-exchange step, which is an advantage compared to the other category of perovskites. The single-step solution-processable characteristics enable quasi-2D perovskite an ideal candidate for large-area device fabrication, because they are more compatible with roll-to-roll or inject printing manufacture.

Fast and homogeneous crystallization is the prerequisite to guarantee high-quality quasi-2D film formation, which is also the most important step in the whole PeLEDs fabrication^20,21^. To date, “antisolvent-assisted” spin-coating was proved to be the most effective approach to fabricate high-quality quasi-2D films^16,21,22^. Basically, antisolvent speeds up precursors to reach the supersaturated state, and subsequently induced rapid and extensive nucleation. Consequently, smooth and dense quasi-2D films with reduced grain size were generated, which greatly eliminates the carrier shunting paths and non-radiative recombination centers. Thus, the accurate addition of antisolvent is vitally important; however, in reality, the time-window for dripping antisolvent is ultra-narrow^23,24^. When the device area is enlarged, whether antisolvent can uniformly diffuse around the large-area substrate in such a narrow time, to initiate the rapid and homogeneous crystallization is questionable. Moreover, antisolvent has been demonstrated to be able to modulate the film’s energy landscape^22^, thereby influencing the film’s emission wavelength and corresponding photoluminescence quantum yield (PLQY). Hence, whether the antisolvent inhomogeneous distribution will cause the optical inhomogeneity is another aspect that needs to be studied. Nevertheless, many factors are still unclear in large-area quasi-2D PeLEDs fabrication. Hence, verifying the validity of the “antisolvent-assisted” approach, exploring the failure mechanism, and seeking new solutions are essential steps for developing high-performance, large-area quasi-2D PeLEDs.

Here, quasi-2D perovskite systems with a composition of PEA_2_(FA_0.7_Cs_0.3_)_*n*−1_Pb_*n*_Br_3*n*+1_ (*n* = 2, 3, …, ∞) are firstly employed to fabricate large-area PeLEDs. However, we find out that the traditional “antisolvent-assisted” approach does not work well for large-area PeLEDs manufacture. Poor film morphology and low PLQY were observed especially in the edge-region of these films. Moreover, the inhomogeneity brings in additional shunting paths, leading to huge leakage current to deteriorate the device. The problems were attributed to severe phase-segregation (2D and 3D phases), since the crystal growth rates and preferable orientation between these two phases are dramatically different. We conclude that the key to generate efficient large-area quasi-2D PeLEDs is to suppress the severe phase-segregation, particularly in the edge-region of the device. Consequently, inhibiting 2D and 3D phases’ precipitation is critical, which can be realized by modulating the intermediate phase to change the crystallization pathway. Accordingly, with the help of the DFT simulation, L-Norvaline (NVAL) is selected to use to construct the new COO^−^-coordinated intermediate phase, which possesses quasi-2D geometry. The new intermediate phase does change the crystallization pathway and facilitate the formation of large-area, high-quality quasi-2D films with desirable optical and electrical properties. We then correlate the film’s emission behavior with their exciton binding energy (*E*_b_) and demonstrate the quasi-2D film’s *E*_b_ needed to be judiciously regulated for the purpose of different PeLED applications. Taking advantage of the above findings, here we reported a series of highly efficient, large-area quasi-2D PeLEDs with active areas of 9.0 cm^2^. The peak EQE of 16.4% has been achieved for PeLED with <*n>* = 3 film, representing the most efficient large-area PeLED so far^13,25–28^. Meanwhile, high brightness device with a luminance up to 9.1 × 10^4^ cd m^−2^ is achieved in <*n>* = 10. Moreover, for small-area devices (0.086 cm^2^), EQE up to 21.3% and luminance up to 1.4 × 10^5^ cd m^−2^ have been realized, which is comparable to the state-of-the-art devices reported to date. The work thus paves the way for the future large-area PeLED manufacture.

## Results

### Large-area quasi-2D films fabricated by the “antisolvent-assisted” approach

**Fig. 1:**
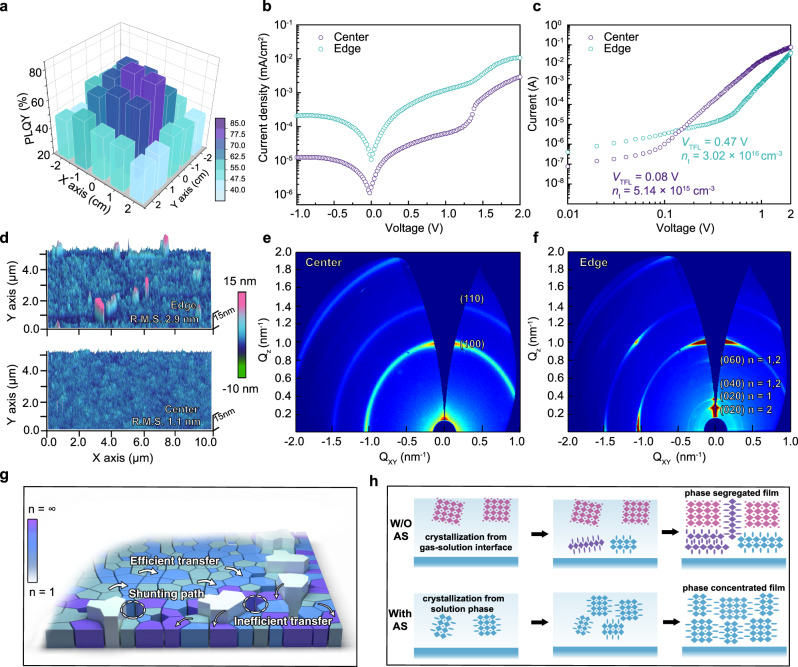
Characterizations of the large-area quasi-2D film fabricated by the “antisolvent-assisted” approach. **a** PLQYs of the large-area quasi-2D film with film area up to 25 cm^2^; PLQY is present using a 5 × 5-pixel fashion to distinguish the center- and edge-region (excitation intensity at ~10 nJ cm^−2^). **b** Current density–voltage (*J–V*) curve for the film at different regions, to illustrate different leakage current levels under reverse bias. **c** SCLC measurements and the extracted defect densities of the film at different regions. **d** AFM images of the film in different regions. GIWAXS patterns of the film at **e** center- and **f** edge-region. **g** Schematic diagram shows the phase-segregation in the edge-region, and the corresponding non-radiative loss pathways. **h** Growth kinetics and phase distribution of quasi-2D films without and with antisolvent.

The “antisolvent-assisted” approach was employed to fabricate large-area <*n>* = 5 quasi-2D, PEA_2_(FA_0.7_Cs_0.3_)_4_Pb_5_Br_16_, perovskite films to verify its validity in large-area device fabrication. Here, <*n*> represents the average number of inorganic layers in the multiple-quantum-well film. Replacing FA^+^ cations with an appropriate amount of Cs^+^ cations has been proved to reduce the grain size and trap density of the films^29^. To realize a high-quality large-area device, the uniformity of both optical and electrical property in the whole film is highly demanded. We thoroughly investigate the film’s physical property, in particular, focusing on the properties difference between center- and edge-area. We first study the film’s PL properties in different regions to evaluate the film’s optical uniformity (Fig. 1a and Supplementary Fig. 1). As shown in Fig. 1a, the PLQY at the center-region exhibits high values that are comparable to the small-area devices^15,21^. However, the PLQY drops rapidly from the center-region to the edge-region, illustrating severe optical inhomogeneity. Transient absorption (TA) measurement was conducted to analyze this optical discrepancy between different regions (Supplementary Fig. 2). We extracted the fast component decay constants (*τ*_1_) of each phase by tracing the bleaching signals in different timescales. As revealed, the decay rates for the center-region are much faster than those for the edge-region, illustrating better energy transfer efficiency, which can be the reason for PLQY enhancement.

We then investigate the electrical properties in different regions. As well known, leakage current under reverse bias is a critical evaluation factor for LEDs. If a large reverse current exists, it means the injected current can flow across the devices without any contribution to light emission. Thus, minimizing the leakage current at a low level is a prerequisite to achieve high EQE and luminance efficiency^30^. To extract the leakage current, large-area quasi-2D PeLED with regular device architecture was fabricated, then been cut into several pieces for characterization. As illustrated in Fig. 1b, an order of magnitude higher leakage current was observed at the edge-region compared to the central one, indicating that additional electrical shunting paths or pinholes exist. Furthermore, a space-charge-limited current (SCLC) technique was utilized to characterize the film’s trap densities (Supplementary Note 1). As shown in Fig. 1c, almost five-times higher trap density was found at the edge sample, demonstrating dramatically different electrical properties between different regions.

To better understand the differences, atomic force microscopy (AFM) was used to examine the surface morphology. As shown in Fig. 1d, perovskite grains are uniformly distributed at the center of the films, with a low root mean square roughness (R.M.S.) of 1.1 nm. In contrast, larger sheet structure together with pin-holes were clearly observed at the edge sample, which results in high surface roughness of 2.9 nm. A similar phenomenon was noticed in the scanning electron microscope (SEM) images too (Supplementary Fig. 3). According to previous reports^31^, the large sheet structure grains can be identified as 2D (*n* = 1) phases coacervate, which was also confirmed by SEM images with different <*n>*-values (Supplementary Fig. 4). Grazing incidence wide-angle X-ray scattering (GIWAXS) measurements further proved our conclusions. As shown in Fig. 1e, Debye–Scherrer rings, (100) and (110) planes, belonging to *n* > 2 quasi-2D species, were observed at high *Q* values space^24^, suggesting the composition at the center-region is dominated by the medium *n* values (*n* > 2). While in the GIWAXS pattern of the edge sample, some Debye–Scherrer rings at low *Q* values were emerged, which ascribed to the (0k0) planes of the *n* = 1 and *n* = 2 species^32^. Moreover, the GIWAXS data are consistent with the X-ray diffraction (XRD) and steady-state UV–vis measurements (Supplementary Fig. 3). Here, strong diffraction peaks and absorption signals ascribed to 3D and 2D phases were clearly observed at the edge sample. Consequently, differences in crystallization rates and preferable orientation between 2D and 3D phases result in the formation of severe phase-segregation and pin-holes, as well as surface roughness. In addition, in the 2D phase, deep defect states will more easily to generate due to the bandgap broadening^33^. These defect states act as charge carrier traps during the energy transfer process, thereby reducing the energy transfer efficiency. Thus, ideally, we expect the quasi-2D phases to be concentrated in the middle *n*-values, rather than 2D and 3D perovskite phases.

### Failure mechanism investigation

We thus conclude phase-segregation should ascribe to the inhomogeneous diffusion of antisolvent, since the antisolvent could not uniformly diffuse to the edge-region in a short time after the substrate area was enlarged. Hence, different crystallization kinetics exist in different regions. To confirm the hypothesis, we first carried out the morphology and optical characterizations of the large-area perovskite films without using antisolvent (Supplementary Fig. 5). The similar morphology and spectroscopy at center- and edge region proved that the evaporation rate difference in different regions is not the main reason causing phase-segregation. Then, we record the XRD pattern evolution as a function of time for the films with and without antisolvent (Supplementary Fig. 6). Only quasi-2D species with the medium *n*-values (*n* > 1) can be found in the case with antisolvent. By contrast, in the case of without antisolvents, the 3D perovskite phase was observed immediately; 2D or quasi-2D perovskite phases emerged much later, which directly lead to phase-segregation. The phenomena are consistent with previous research^34–36^. The phase-segregation not only deteriorated the film’s electrical properties, but also decreased the emitting efficiency due to the inhibited energy transfer, as illustrated in Fig. 1g.

We attributed the diverse crystallization behaviors arising from chemical components’ spatial distribution difference between surface and interior of the precursor, as well as the supersaturation degree difference between center- and edge-region. According to the classical LaMer model^37^, in principle, the nucleation and subsequent crystal growth take place when the monomer concentration reaches the minimum nucleation concentration, then driven by the degree of supersaturation. As shown in Fig. 1h, when in the absence of antisolvent, due to the evaporation of the solvent molecules on the surface, the precursor solution at the liquid-solution interface first reaches a supersaturated state and crystallizes. This top–down crystallization mode further leads to a gradient distribution of phases in the quasi-2D film^38^. Since large-volume organic ions (BA^+^/PEA^+^) tend to remain in the solution, at the initial period of the nucleation, more Cs^+^ or FA^+^ ions concentrated on the surface of the solution to maintain electric neutrality. Thus, inorganic slabs released from the precursor-solvent complexes are consequently combined with more Cs^+^/FA^+ 39^, resulting in abundant large *n* or 3D perovskite phases. Then, as the accumulation of large organic cations, 2D phases are subsequently generated. On the contrary, once antisolvent dripped, the precursors rapidly reached a high level of supersaturation, resulting in spatial uniformly distributed nuclei. These nuclei then initiate quasi-2D crystal growth, leading to homogeneous and narrow *n*-value distributed quasi-2D films.

Inspired by the above findings, we conceive to explore a new quasi-2D intermediate phase with even lower nucleation barrier. The quasi-2D intermediate phase should facilitate the succeeding quasi-2D crystal growth and suppress the 3D one. Anion engineering was proved to be an effective approach to modulate intermediate phases, thereby manipulating the crystallization. Particularly, carboxylate anion (COO^−^), as a bidentate ligand, exhibits a strong binding affinity to perovskite precursors, which will generate a new intermediate phase^40,41^. Basically, the bond between COO^−^ and Pb^2+^ is the coordinate bond, which enables COO^−^ to permanently occupy the X-site of resulting perovskites. Thanks to the strong strength of covalent bonds, the new COO^– ^coordinated intermediate phase should possess relatively lower formation enthalpy in principle. That means the new COO^−^-containing intermediate phase has priority over 3D phase to precipitate in the crystallization. Following this trend, with the help of DFT simulation, dipolar amino acid that contains both COO^−^ and NH_3_^+^ groups was selected. This is because the NH_3_^+^ groups will enable newly formed COO^−^-coordinated intermediate phase to adopt quasi-2D geometry.

### Intermediate phase modulation

**Fig. 2:**
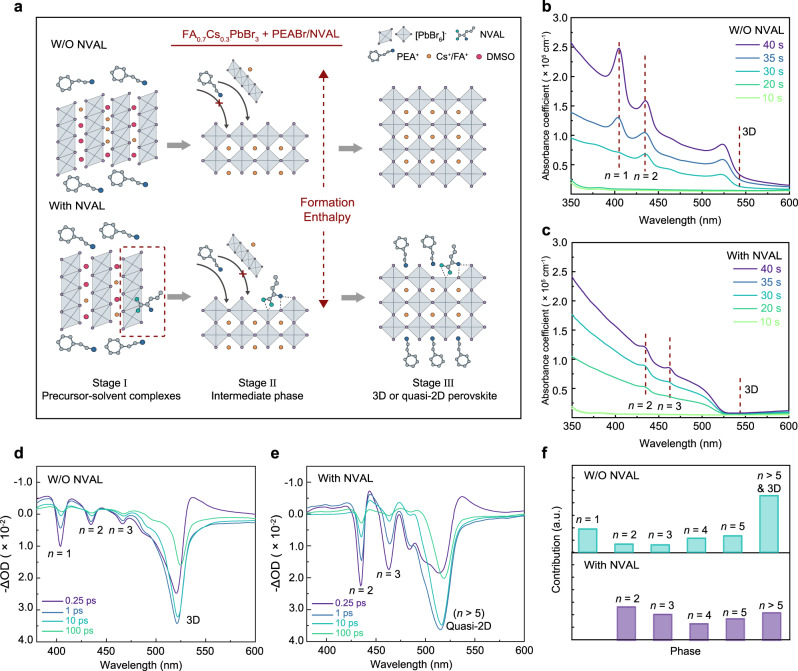
Modulate the intermediate phase via NVAL additive, to suppress the phase-segregation. **a** Schematic diagram for nucleation and growth processes of quasi-2D perovskite without and with NVAL. DFT simulation indicated the NVAL surface-anchored intermediate phase possesses lower formation enthalpy. **b**, **c** Steady-state UV–vis spectra at different spin-coating times to probe the perovskite phase evaluation of the quasi-2D perovskite films without and with NVAL. **d**, **e** TA spectra at selected timescales of the quasi-2D perovskite films without and with NVAL. **f** Relative contents of different phases for quasi-2D perovskite films without and with NVAL, the relative contents were obtained according to the amplitude of GSBs in TA spectra at 0.25 ps.

Here, a short-chain α-amino acid, L-Norvaline (NVAL), was selected, since the molecular configuration of NVAL allows NH_3_^+^ and COO^−^ groups to occupy adjacent A- and X-site of the perovskite intermediate phase at the same time. Figure 2a illustrated the schematic diagram for the new crystallization pathway after introducing the NVAL additive. The NVAL firstly coordinates with [PbBr_6_]^4−^ inorganic slabs in the precursor status, then as the evaporation of solvent, NVAL incorporates within the quasi-2D crystal as an organic spacer. Formation enthalpies were simulated (Supplementary Note 2 and Supplementary Fig. 7) to evaluate whether the COO^−^-coordinated intermediate phase with quasi-2D geometry is favorable to form. As shown, once NVAL anchored on the perovskite surface, formation enthalpy for this new intermediate phase was determined to be −1.258 eV. The value is much lower than traditional PEA-anchored quasi-2D intermediates (−0.242 eV), indicating the newly formed intermediate phase is thermodynamically more favorable. In experiments, coordination between NVAL and [PbBr_6_]^4−^ inorganic slabs was confirmed in Fourier transform infrared (FTIR) and ^1^H nuclear magnetic resonance (NMR) spectroscopy (Supplementary Fig. 8). In specific, the red-shift of C=O stretching vibration peak, and downfield-shift of proton resonance signals were clearly observed.

Along with the solvent evaporation, crystal growth upon the nuclei gradually take place, which denotes as stage II and III (Fig. 2a). As shown, once the NVAL coordinated intermediate phase emerged, the resulting steric hindrance inhibits the perovskite growth towards 3D configuration. To confirm the findings, <*n>* = 5 quasi-2D films were fabricated. Steady-state UV–vis spectra at different times were recorded to monitor the phase evolution (Fig. 2b, c). Without the NVAL, *n* = 1, *n* = 2, as well as 3D phase clearly appeared in the film. Moreover, 3D phase emerged prior to the 2D and quasi-2D phases as shown, further demonstrating phase-segregation arose from nucleation barrier differences. In contrast, in the NVAL-treated sample, quasi-2D phases appeared almost at the same time, and the undesirable 3D or 2D phase disappeared. The phenomenon implies that phase-segregation has been substantially inhibited.

TA measurements were employed to detect the *n*-value distribution and resulting energy transfer^13,21^. Corresponding TA spectra are shown in Fig. 2d, e. As revealed, the sample without NVAL exhibits several dominated ground-state bleach (GSB) peaks, which can be assigned as *n* = 1, 2, 3, and 3D phases. However, the film fabricated with NVAL additive displays characteristic quasi-2D GSB peaks, but in the absence of *n* = 1 and 3D phases. Based on the amplitude of initial GSB peaks, we can qualitatively evaluate the relative contents of each *n* value species^21^. As shown in Fig. 2f, *n* = 1 and 3D phases dominated in the film without NVAL, while the film with NVAL manifests features of narrow *n*-value distributed quasi-2D perovskites. We then estimate the energy transfer kinetics for these two samples (Supplementary Fig. 9). As shown, NVAL-containing film shows more efficient energy transfer compared to the film without NVAL, as it possesses a better-graded energy landscape. To explore whether other *α*-amino acids also have a phase distribution regulation effect, we also introduced other additive molecules (Supplementary Table 1 and Supplementary Fig. 10). Unfortunately, due to the long NH_3_^+^-COO^−^ distance and limited solubility of these amino acids, PLQY of the films and devices’ EQEs were still low.

### Characterizations of the resulting large-area quasi-2D films

**Fig. 3:**
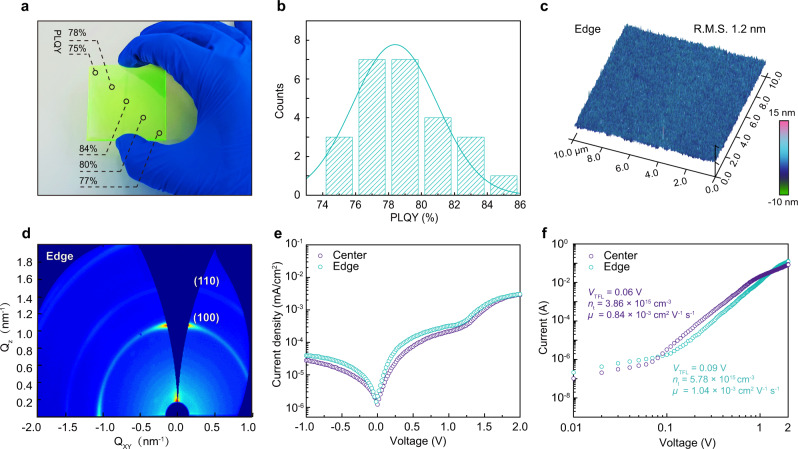
Characterizations of the large-area quasi-2D film. **a** Digital photo of the resulting large-area quasi-2D perovskite film and the corresponding PLQY in select regions. **b** Statistical PLQY for the 25 pieces films, which originate from different regions of the large-area quasi-2D film. **c** AFM image and **d** GIWAXS pattern of the film at the edge-region. **e** *J*–*V* curves of the film at different regions, to extract the leakage current level under reverse bias. **f** Different region film’s SCLC curves and the corresponding defect densities.

The suppressed phase-segregation encouraged us to explore this NVAL-treated approach into large-area quasi-2D film fabrication. Thus, a 5.0 × 5.0 cm^2^ large-area film was fabricated. As shown in Fig. 3a, the corresponding quasi-2D film exhibits uniform and bright emission under ambient conditions. The film was then cut into 25 pieces to investigate emitting properties, respectively. As shown, the film exhibits high PLQY with small variations among different pieces, with the average PLQY value of ~80% (Fig. 3b). In addition, the PL emission centered at 525 nm with a narrow half-peak width of 21 nm (Supplementary Fig. 11) for all samples, demonstrating excellent optical homogeneity.

Morphology and electrical property were then thoroughly investigated by AFM and SEM characterizations (Fig. 3c and Supplementary Fig. 11). The resulting films displayed dense and smooth morphology, particularly possessing a low R.M.S. of 1.2 nm at the edge-region. TEM measurements also indicated the significantly reduced grain size in the NVAL-containing film (Supplementary Fig. 12). The improved film quality was ascribed to the elimination of phase-segregation, since no 2D and 3D phases have been observed. The corresponding phase distribution was further characterized by the GIWAXS measurement (Fig. 3d). In specific, no Debye–Scherrer rings at low *Q* values can be observed in the edge-region sample, indicating the absence of 2D phase, which is consistent with the TA data. Consequently, fairly low leakage currents were observed for the whole film, indicating that electrical shunting paths that previously exist in the edge-region were eliminated (Fig. 3e). In addition, SCLC measurements show reduced trap density over the entire film (Fig. 3f), in agreement with other characterizations.

### Recombination kinetics for quasi-2D films with different *E*_b_

The high-quality large-area quasi-2D film lays the foundation for high-performing large-area PeLEDs realization. However, beyond the film quality, the electroluminescence (EL) behavior is strongly dependent on the material’s carrier recombination characteristics. Especially for devices with different application purposes, such as high current efficiency or high-brightness, a deep understanding of the carrier recombination kinetics and then regulating the emission behavior is highly demanded. Generally speaking, efficient exciton recombination rate facilitates yielding high PLQY, meanwhile, the exciton recombination rates positively correlated to the material’s *E*_b_^4,42^. However, this does not mean a film possessing high *E*_b_ is applicable for realizing high-performance devices, because the *E*_b_ is also highly related to the Auger recombination process^43^. Auger recombination will cause efficiency roll-off under high current density, significantly hindering the LEDs to achieve high brightness^44^. Therefore, the *E*_b_ should be judiciously selected. The structural tunability of quasi-2D perovskite offers an opportunity to tune their *E*_b_ and corresponding carrier recombination kinetics. As is well known, *E*_b_ of quasi-2D perovskites increases with decreased quantum-well thickness^45^. Basically, by adjusting <*n>*-values, the corresponding quasi-2D perovskite films’ emitting behavior can be regulated.

**Fig. 4:**
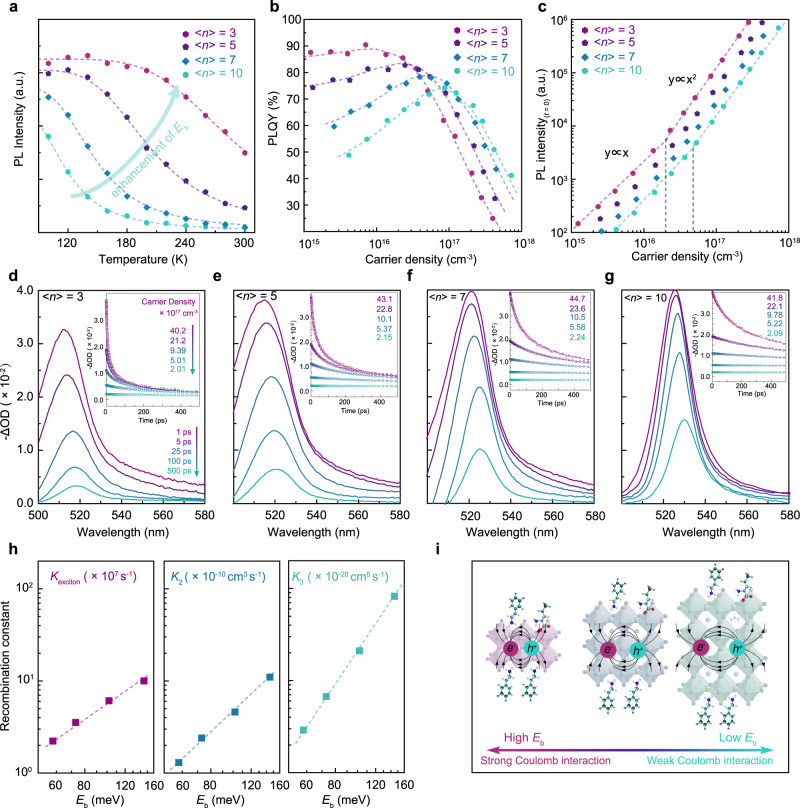
Recombination kinetics of the corresponding quasi-2D films with different *E*_b_. **a** Temperature-depended PL measurements for different <*n>-*value quasi-2D films to characterize the corresponding *E*_b_. **b** Power-dependent PLQY and corresponding **c** PL intensity (*t* = 0) as a function of initial carrier density for different quasi-2D films. **d**–**g** TA spectra at selected timescales under carrier density around 4 × 10^18^ cm^−3^ for different quasi-2D films. Inset: evolution of TA bleaching decays as a function of carrier density on a 500 ps timescale. **h** The extracted recombination constants through global fitting as a function of *E*_b_. **i** Schematic diagram shows the electron–hole Coulomb interaction in quasi-2D perovskite with different quantum well thicknesses.

To further understand the relationship between material’s *E*_b_ and corresponding carrier recombination kinetics, we fabricated quasi-2D films, PEA_2_(FA_0.7_Cs_0.3_)_*n*-1_Pb_*n*_Br_*3n*+1_, with <*n>* = 3, 5, 7, and 10. Morphology and SCLS measurements as well as optical characterizations indicated that all films display high uniformity without any noticed phase-segregation (Supplementary Figs. 13–18), demonstrating the versatility of this NVAL-treated approach. We extracted the *E*_b_ of different <*n>*-values quasi-2D films from temperature-dependent PL spectra and found the *E*_b_ increases from 58 to 152 meV, when the <*n>*-values decrease from 10 to 3 (Fig. 4a and Supplementary Fig. 19). We then carried out the power-dependent PLQY measurements to investigate their PL characteristics. As shown in Fig. 4b, the <*n>* = 3 film yielded a peak PLQY up to 91% under a low excitation intensity of ~6.0 × 10^15^ cm^−3^; and no PLQY decline was observed when excitation density is lower than 10^16^ cm^−3^. Whereas, as the <*n>*-values increased, the maximum PLQY decreased, indicating overall lower radiative recombination efficiency. Moreover, all the films exhibit PLQY roll-off under high excitation density, and the observed roll-off thresholds increased with increasing <*n>*-values.

According to the recombination mechanism, the carrier recombination in the perovskite film is actually the sum of monomolecular, bimolecular, and Auger recombination (Supplementary Note 3)^42,45,46^. Generally, *k*_1_, *k*_2_, and *k*_3_ are defined as recombination constants of monomolecular, bimolecular, and Auger recombination, respectively. While in quasi-2D perovskites, monomolecular recombination constant *k*_1_ represents the sum of radiative exciton recombination (*k*_exciton_) and nonradiative trap-assisted recombination (*k*_trap_)^47^. Thereby, the high PLQY achieved in low <*n>*-value films can be understood as rapid exciton recombination outcompete the trap-assisted recombination. Power-dependent PL_*t* = 0_ measurements further identified the hypothesis (Fig. 4c). Under low carrier densities, first-order dependence of PL_*t*=0_ intensity on excitation intensity reveals that exciton recombination is dominated here. As the carrier density further increases, the dependence of the PL intensity on excitation intensity gradually changes to second-order in all the films. This is because the exciton tends to dissociate into free carriers under high excitation intensity^47^. Accordingly, relatively lower thresholds of first-order to second-order transition observed when <*n>*-values decreased, illustrating the enhanced *k*_2_ with increasing <*n>*-values. The subsequent PLQY roll-off is attributed to the Auger recombination. As noticed, small PLQY roll-off thresholds for low <*n>*-value films suggested that they possess higher *k*_3_.

The above results revealed that there is a strong correlation between emission behavior with *E*_b_. To investigate the underlying relationship intuitively, recombination rate constants were then extracted. In brief, *k*_1_ was extracted from the slow decay of time-resolved PL (TRPL) under low excitation intensity (Supplementary Fig. 20). Then, *k*_2_ and *k*_3_ were obtained from the “global” fitting of TA data (Supplementary Note 3), which was finished by fitting the bleaching signals at different excitation intensities, as displayed in Fig. 4d–g. TA kinetics measurement at different regions confirmed that the perovskite films at the center- and edge-region share similar recombination behavior (Supplementary Figs. 21 and 22). As mentioned, the extracted *k*_1_ here actually is the sum of *k*_exciton_ and *k*_trap_; thus, power-dependent PLQY was fitted to further distinguished *k*_trap_ and *k*_exciton_. The corresponding recombination rate constants were summarized in Supplementary Table 2. Near-linear (~1.3 order) increment of *k*_exciton_ with *E*_b_ can be found in Fig. 4h. Usually, the recombination rate constants were proportional to the Coulomb force between electron and hole^48^. Thus, we conclude the upward trend is caused by Coulomb interaction enhancement. As the <*n>*-value decreased, the phase distribution concentrated in the domains with small *n* values, thus improving the confinement effect. The strong quantum confinement limited spatial extension of the wave function. In addition, reduced <*n>*-values also weakened the dielectric shield^49^. Both quantum and dielectric confinement increase the electron–hole Coulomb interaction and *E*_b_ (Fig. 4i), thus enlarging the probability of electron–hole recombination. Furthermore, nonlinearity dependencies of ~2.2 and 3.3 order for *k*_2_ and *k*_3_ revealed different sensitivities of recombination constants to *E*_b_. This can be explained by the enhanced electron–hole Coulomb interactions and their bimolecular or three-body recombination characters (Supplementary Note 3). Notably, strong Coulomb interaction will induce unbalanced electron–hole distribution, which aggravated the undesired *eeh* or *hhe* Auger recombination^50^.

The tunable emission behavior thus enables quasi-2D perovskite films suitable for PeLEDs fabrication, particularly for the purpose of different applications. Low <*n>*-value films possess rapid exciton and free carrier recombination which avoid the carriers dissociating and quenching in the trap states. As a result, low <*n>*-value films exhibit high PLQY under a wide range of injected current. These films exhibit advantages in display applications where high current efficiency under low current density is particularly required. However, the low <*n>*-value films cannot reserve high PLQY under high current density because of the effective Auger recombination, thus limiting their application in high-brightness required devices, such as solid-state lighting. Fortunately, high <*n>*-value analogs with lower *E*_b_ emerge as promising candidates. High <*n>*-value films possess improved conductivity for better injection and transport; more importantly, lower PLQY roll-off was observed thanks to the reduced Auger recombination rates. Hence, higher <*n>*-value quasi-2D PeLEDs should be able to achieve high-brightness, together with moderate EQE in principle.

### High-performing large-area quasi-2D PeLEDs

**Fig. 5:**
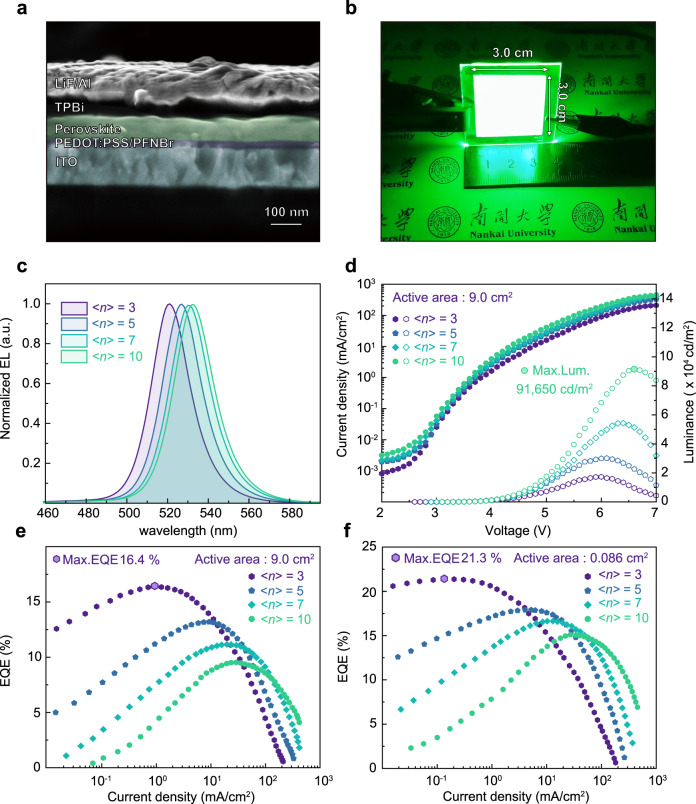
Characterizations of large-area and small-area PeLEDs of quasi-2D perovskite films with different <*n>-*values. **a** Cross-sectional SEM image of the corresponding PeLED device. **b** Digital photo of the large-area PeLED device operated under biased voltage of 4.5 V (<*n>* = 3), with active areas of 9.0 cm^2^. **c** Normalized EL spectra for different <*n>*-value devices. **d** Current density–luminance–voltage *(J–L–V)* and **e** EQE–current density *(EQE–J)* characteristics of large*-*area PeLEDs, with an active area of 9.0 cm^2^. **f** *EQE–J* curves of small-area PeLEDs, with an active area of 0.086 cm^2^.

Thus, to validate our findings, we fabricated large-area quasi-2D PeLED with an active area of 9.0 cm^2^ adopting ITO/PEDOT:PSS/PFNBr/Perovskite/TPBi/LiF/Al device architecture. Here, an ultrathin PFNBr interface layer was inserted, to improve the wettability and minimize the interfacial recombination, based on the previous reports^51,52^. Cross-sectional SEM images reveal the presence of each layer and their thickness (Fig. 5a). The thickness of PEDOT:PSS, PFNBr, quasi-2D perovskite, TPBi, and LiF/Al are 25 nm, <5 nm, 80 nm, 40 nm, and 100 nm, respectively. The energy level diagram of perovskite LEDs was demonstrated through ultraviolet photoemission spectroscopy (UPS) measurements (Supplementary Figs. 23 and 24). Figure 5b shows a photo of the large-area device under operating conditions. Uniform EL emission was observed over the entire device, indicating high optical and electrical uniformity. The EL peak of the <*n>* = 10 device centers at 534 nm; continuous blue shifts were observed as <*n>*-value decreases, <*n>* = 3 displays an EL peak located at 520 nm (Fig. 5c). All of the devices display improved Commission Internationale de I’Echlaiage (CIE) color coordinates compared to the National Television System Committee (NTSC) standard, which is very close to the most advanced Rec.2020 display standard (Supplementary Fig. 25).

The current density–luminance–voltage *(J–L–V)* and EQE–current density *(EQE–J)* curves (Fig. 5d, e) exhibit different EL behaviors of the devices. As expected, the resulting EQE for different <*n>*-values is in good agreement with the power-dependent PLQY data. The corresponding <*n>* = 3 device delivered a peak EQE of 16.4% at a low current density of 1.0 mA cm^−2^, representing the most efficient large-area PeLEDs to date (Supplementary Tables 3 and 4). The <*n>* = 3 device displayed fairly high EQE under low current density, demonstrating its great potentials for large-area display. With the increase of <*n>*-values, a higher threshold for EQE roll-off was achieved. The <*n>* = 10 device thus shows a maximum luminance of 91,650 cd m^−2^, demonstrating its potential in high-brightness lighting. Moreover, the devices show high EL uniformity under different constant voltages (Supplementary Fig. 26).

The PeLED performances with various active areas were also studied and summarized (Supplementary Figs. 27–29). As shown, a maximum luminance of 143,410 cd m^−2^ was obtained in small-area <*n>* = 10 device, with an active area of 0.086 cm^2^. Moreover, a high EQE of 21.3% was achieved in <*n>* = 3 device (Fig. 5f), which is comparable to the state-of-the-art PeLEDs reported to date (Supplementary Tables 5 and 6). The device incorporated with NVAL also exhibited an appreciable enhancement in operating stability (Supplementary Figs. 30 and 31). Moreover, our strategy also shows good compatibility with the spin-coating technology (Supplementary Fig. 32), demonstrating its universality for large-area PeLEDs fabrication.

## Discussion

In summary, we have developed an “antisolvent-free” method to fabricate large-area quasi-2D perovskite films, which is achieved by partially anchoring amphiphilic NVAL molecules on [PbBr_6_]^4−^ inorganic slabs. Simulation and experimental characterizations confirm that the surface anchoring of NVAL facilitates a low-barrier crystallization pathway by generating a new intermediate phase with quasi-2D geometry. The approach overcomes the phase-segregation, enabling high-quality large-area quasi-2D films becomes possible. We further thoroughly investigate the carrier recombination kinetics to better understand the film’s recombination features. Basically, the film’s emission behavior highly correlated with their *E*_b_, indicating that *E*_b_ needs to be judiciously selected for the purpose of different PeLED applications. Taking advantage of the above findings, we achieved a series of highly efficient, large-area quasi-2D PeLEDs with active areas of 9.0 cm^2^. The peak EQE of 16.4% has been achieved in <*n>* = 3 films, which is the most efficient large-area PeLEDs to date. Meanwhile, a high brightness device with luminance up to 9.1 × 10^4^ cd m^−2^ has also been realized in <*n>* = 10 perovskite. The work demonstrates quasi-2D perovskites’ great potential and diversity for display or lighting application, thus it paved the way for future large-area PeLEDs manufacture.

## Methods

### Materials

Formamidinium bromide (FABr) and phenethylamine bromide (PEABr) were purchased from Greatcell Solar. CsBr, PbBr_2_, and NVAL were purchased from Sigma-Aldrich. Tetrabutylphosphonium tetrafluoroborate (Bu_4_PBF_4_) was purchased from J&K chemical. Poly(3,4-ethylenedioxythiophene):poly(styrenesulfonate) (PEDOT:PSS) (Clevios P VP Al4083) was purchased from Heraeus. Poly[(9,9-bis(3’-((N,N-dimethyl)-N-ethylammonium)-propyl)-2,7-fluorene)-alt-2,7-(9,9-dioctylfluorene)] (PFNBr), (1,3,5-benzinetriyl)-tris(1-phenyl-1-H-benzimidazole)) (TPBi) and LiF were purchased from Lumtech Corp. All the chemical materials were directly used without any further purifications.

### Precursor solution fabrication

Perovskite precursors were dissolved in dimethyl sulfoxide (DMSO) with 0.4 M (concentration of Pb). For NVAL-containing precursors, 8.0 mol% of NVAL with respect to the lead atoms were also added. Detailed recipes for different precursor solutions were documented in Supplementary Table 6.

### Device fabrication

PEDOT:PSS diluted with deionized water and ethanol (1:1:1, volume ratio) was spin-coated onto ITO for 1 min at 4000 r.p.m., and then annealed for 20 min at 150 °C to remove any residues. PFNBr dissolved in methanol with the concentration of 0.5 mg/mL was then spin-coated on top of the PEDOT:PSS layer for 1 min at 4000 r.p.m. To fabricate the active layers, precursor solutions were spin-coated in glove-box for 1 min at 6000 r.p.m., and then annealed for 10 min at 80 °C. For the “antisolvent-assisted” approach, chlorobenzene as antisolvent was dripped onto the substrate after spin coating for 15 s. Finally, TPBi (40 nm), LiF (0.8 nm), and Al electrode (150 nm) were deposited on the top of the devices through thermal evaporation under a vacuum of <1 × 10^−4^ Pa.

### Film characterizations

The steady-state PL spectra were measured by using a spectrofluorometer (Fluoromax 4, Horiba) with a 450 W Xe lamp. The PLQY were measured using an integrating sphere method, a 365-nm laser was used to photo-excite the films, and a Quanta-Phi integrating sphere with a Fluorolog system was used to collect the spectra. The TRPL spectra were measured by using a spectrometer (FLS980) with a picosecond pulsed laser under a repetition rate of 800 kHz at 355 nm. The time-resolved signal was recorded by a time-correlated single photon counting (TCSPC) module, the total instrument response function (IRF) was less than 100 ps. The PLQY and TRPL at different excitation intensities were recorded at the same external conditions. Temperature-dependent PL spectroscopic measurements were performed in a Horiba, LabRAM HR 800 equipment, the sample was mounted in a helium cryostat (Linkam), and a 325-nm laser with an excitation power of 3 μW was used to excite the sample. The steady-state absorbance spectra were measured by using a LAMBDA 950 UV/Vis/NIR spectrophotometer. To monitor the phase evolution of the perovskite films, the perovskite film was spin-coated at a low revolving speed of 3000 r.p.m. Transient absorption measurements were conducted by using an Ultrafast Systems LLC pump-probe system. Coherent, 35 fs and 1 kHz ultrashort pump pulse at 800 nm was generated in a multipass femtosecond amplifier system, and a pump wavelength of 365 nm was generated in an optical parametric amplifier (TOPAS-800-fs). White continuum probe pulse was generated by focusing part of the amplifier output onto a constantly rotating CaF_2_ with ~1 mm beam size and ~10 μJ power control. FTIR spectra were recorded under attenuated total reflectance (ATR) mode on a Nicolet iS50 spectrometer. ^1^H NMR spectra were recorded in DMSO-d6 solvent by using a Bruker 400 MHz NMR spectrometer. XRD patterns were obtained using a Bruker D8 X-ray diffractometer (Cu Kα, *λ* = 1.5406 Å, 40 kV, 100 mA). GIWAXS patterns (*λ* = 1.54 Å, incident angle 0.15°) were measured on beamline 1W2A at Beijing Synchrotron Radiation Facility (BSRF), China. AFM images were obtained by using a Bruker dimension icon microscope in a non-contact mode. SEM images were obtained by using a field emission Scanning Electron Microscopy (JSM-7500F, JEOL). The TEM images of the perovskite grains were captured on a TEM machine (Talos F200X G2, FEI). The UPS spectra were recorded using an Omicron ultrahigh-vacuum chamber with excitation provided by the He I emission line (21.2 eV) of a helium pressure about 2 × 10^−8^ mbar.

### Device characterizations

All PeLEDs were tested in a glovebox without encapsulation. The *J–V* characteristics were obtained by using a Keithley 2400 source meter (step size 0.1 V, step interval 0.1 s), and the EL spectra were collected by using an integrating sphere (OceanOptics FOIS-1) coupled to a spectrophotometer (QE65 Pro). All of the EL data was double-checked by a PR-735 spectroradiometer (Photo Research).

## Supplementary information

Supplementary Information

## Data Availability

The data that support the finding of this study is available from the corresponding author upon reasonable request.
