# Supplementary material for: High-performance large-area quasi-2D perovskite light-emitting diodes
Source: Nat Commun. 2021 Apr 13;12:2207. doi: 10.1038/s41467-021-22529-x (PMC8044177; doi:10.1038/s41467-021-22529-x)
Supplement: Supplementary file 1 — Supplementary Information [file 41467_2021_22529_MOESM1_ESM.pdf]

Supplementary Information

High-performance large-area quasi-2D perovskite light-emitting diodes

Changjiu Sun[1,†], Yuanzhi Jiang[1,†], Minghuan Cui[2], Lu Qiao[3], Junli Wei[1], Yanmin Huang[1], Li Zhang[1], Tingwei He[1], Saisai Li[1], Hsien-Yi Hsu[4], Chaochao Qin[2], Run Long[3] & Mingjian Yuan[1,*]

[1] Key Laboratory of Advanced Energy Materials Chemistry (Ministry of Education), Renewable Energy Conversion and Storage Center (RECAST), College of Chemistry, Nankai University, 300071, Tianjin, P. R. China.

[2] Henan Key Laboratory of Infrared Materials and Spectrum Measures and Applications, School of Physics, Henan Normal University, 453007, Xinxiang, P. R. China.

[3] Key Laboratory of Theoretical & Computational Photochemistry of Ministry of Education, College of Chemistry, Beijing Normal University, 100875, Beijing, P. R. China.

[4] School of Energy and Environment & Department of Materials Science and Engineering, City University of Hong Kong, 999077, Hong Kong, P. R. China.

[†] These authors contributed equally to this work.

[*] Corresponding author. E-mail: yuanmj@nankai.edu.cn

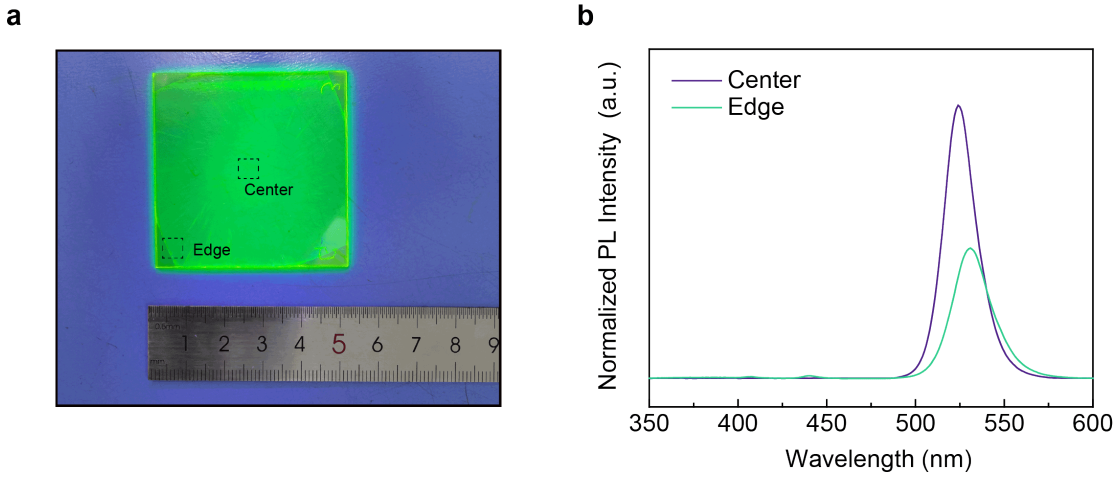

Supplementary Figure 1 | PL measurements of large-area quasi-2D film fabricated by "antisolvent-assisted" approach. (a) Photograph of the quasi-2D film (<*n*> = 5) under UV illumination. **(b)** Steady-state PL spectra of the quasi-2D film (<*n*> = 5) at different regions.

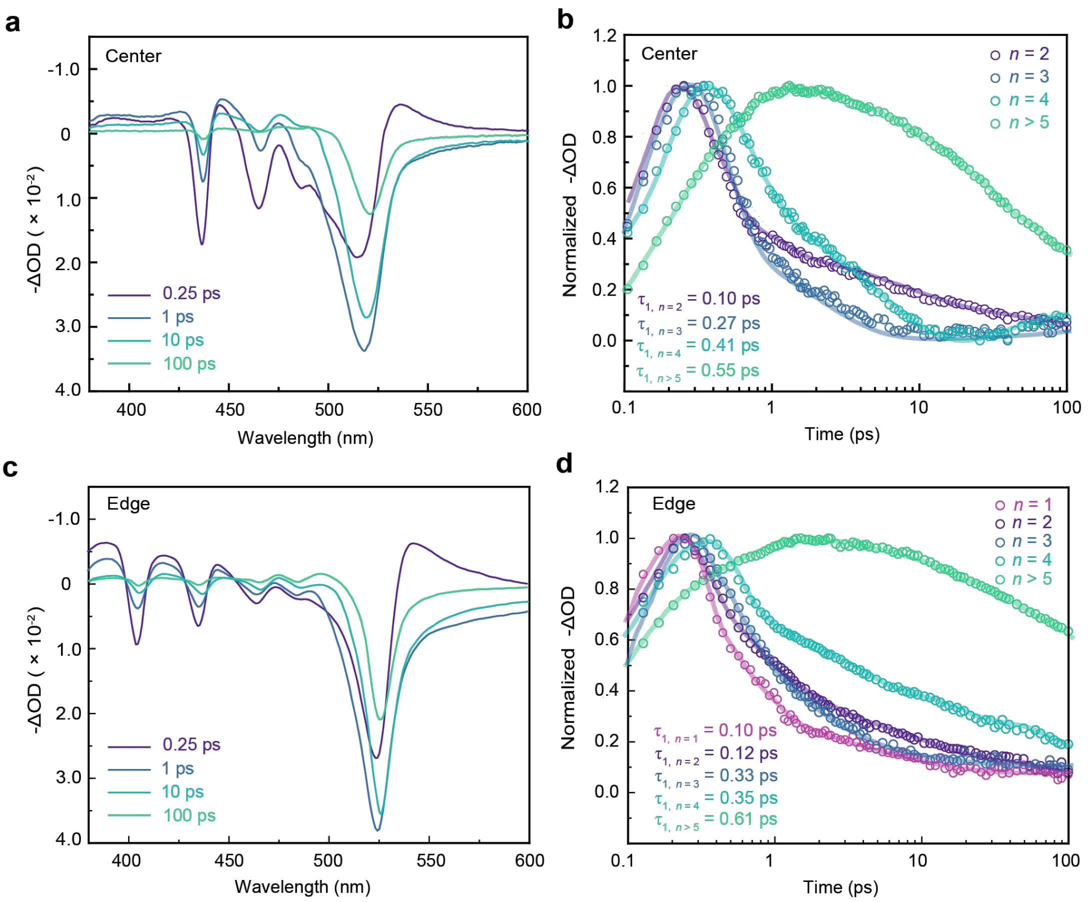

Supplementary Figure 2 | TA data of large-area quasi-2D film fabricated by "antisolvent-assisted" approach. (a, c) TA spectra at selected timescales, and **(b, d)** TA traces as a function of decay time and extracted fast component decay constants (τ_1) for different phases at the center- and edge-region.

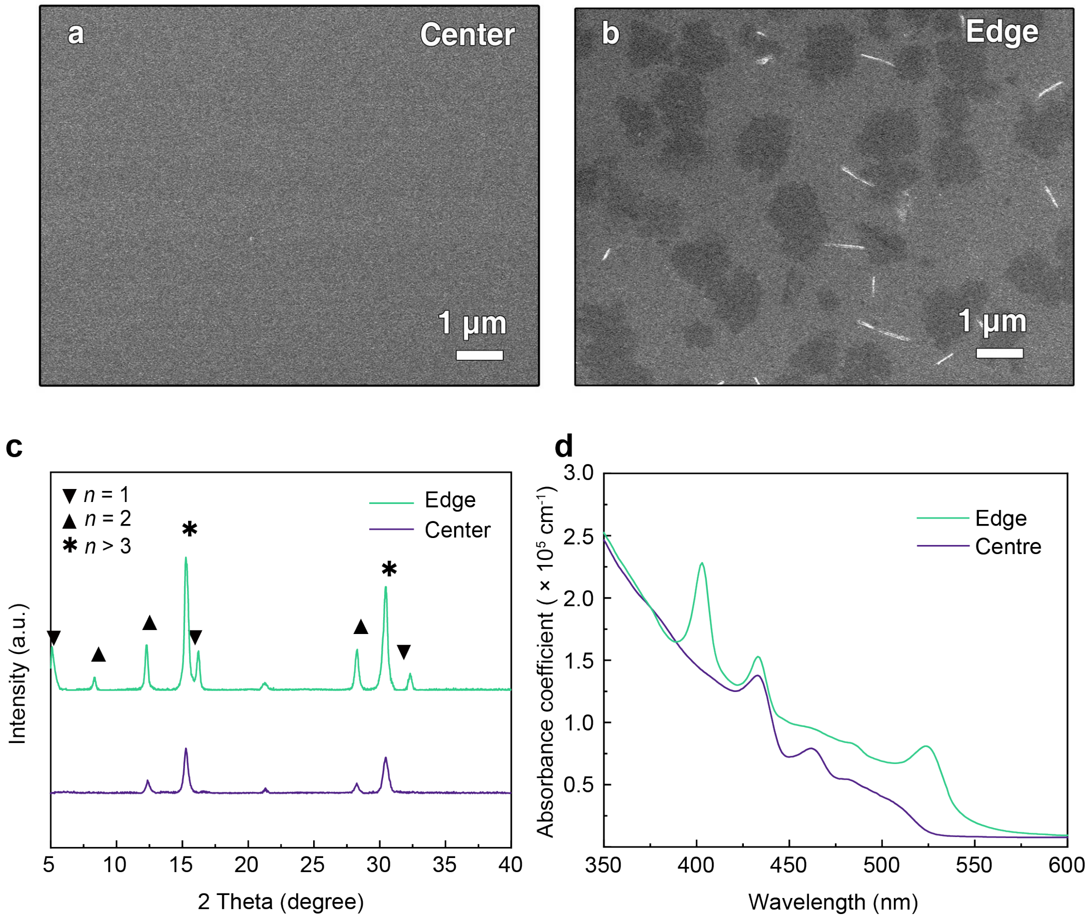

Supplementary Figure 3 | Morphological and spectroscopic measurements of large-area quasi-2D film fabricated by "antisolvent-assisted" approach. SEM images for **(a)** center- and **(b)** edge-region. **(c)** XRD patterns and **(d)** steady-state UV-vis spectra at the center- and edge-region.

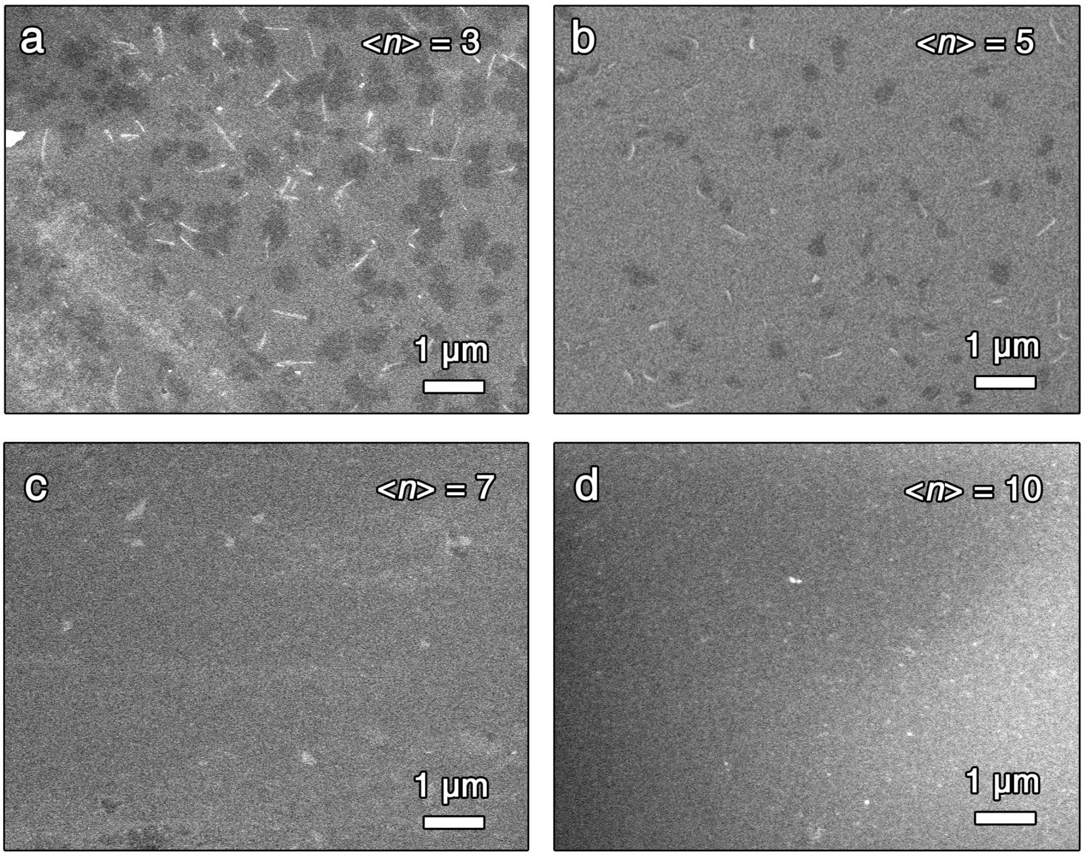

Supplementary Figure 4 | SEM images of the perovskite films with different <*n*>-values without using antisolvent. As the <*n*>-value decreases, the sheet structures in the film gradually increases. This indicates that the sheet structures are more likely to be aggregates of low-*n* or 2D phase.

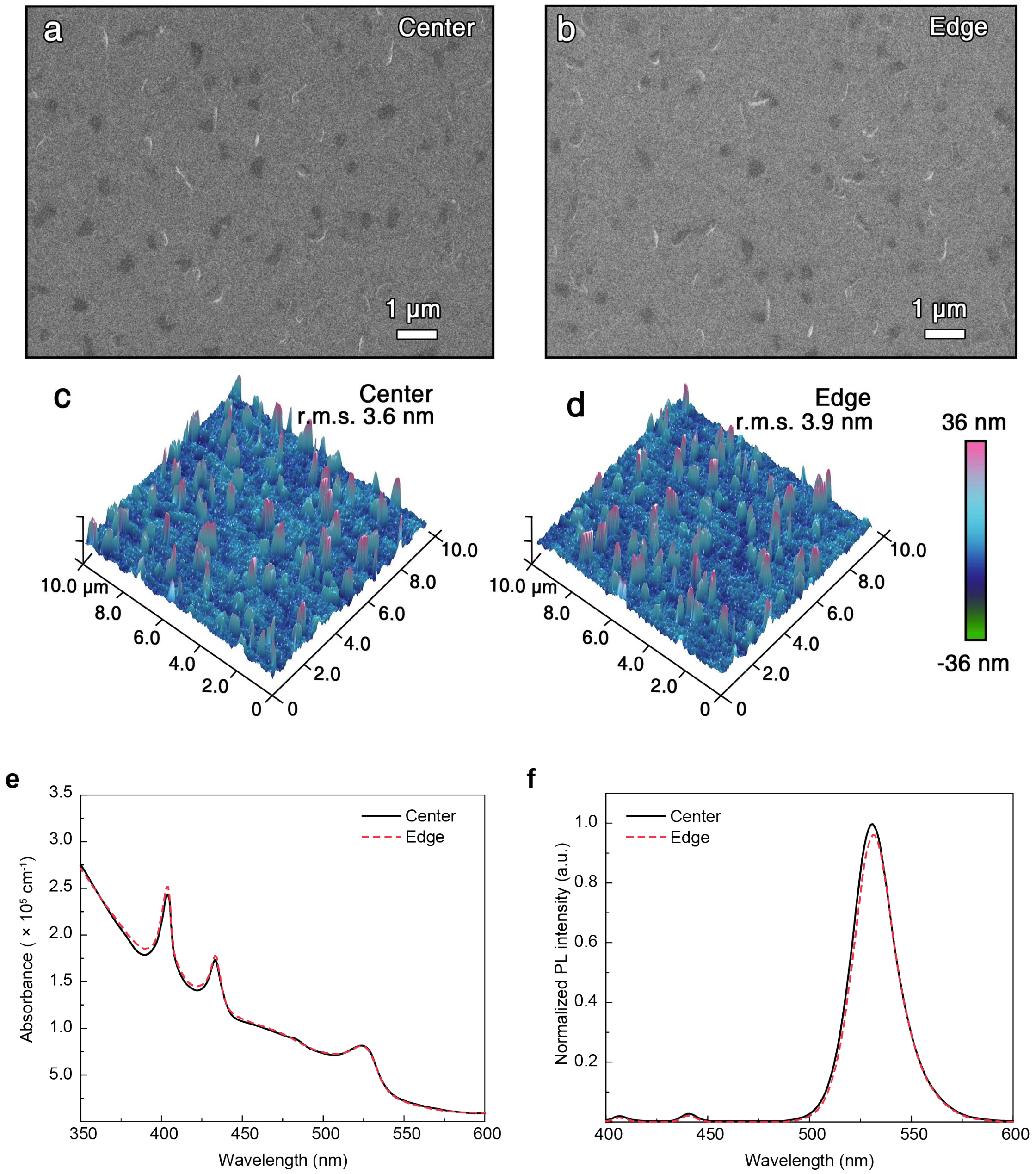

Supplementary Figure 5 | Morphology and optical characterizations for the perovskite films without using antisolvent (<*n*> = 5). (a, b) SEM and (**c, d)** AFM measurements of quasi-2D films. **(e)** steady-state UV-vis and **(f)** steady-state PL spectra of the quasi-2D films.

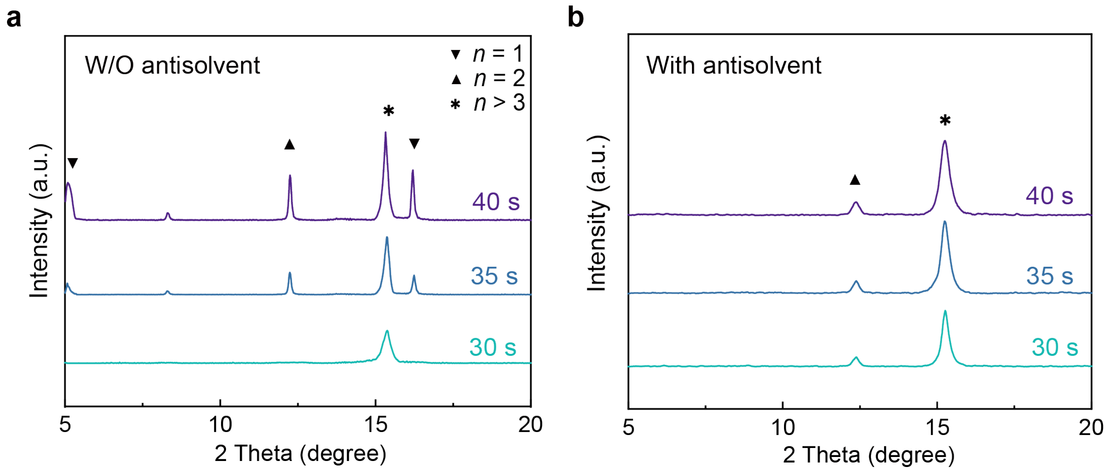

Supplementary Figure 6 | XRD patterns of quasi-2D perovskite films. Quasi-2D perovskite films (<*n*> = 5) fabricated **(a)** without and **(b)** with antisolvent. The evolution of the XRD pattern shows that the generation of the quasi-2D or 2D phase with a substantial delay with respect to the 3D phase, when antisolvent was absent.

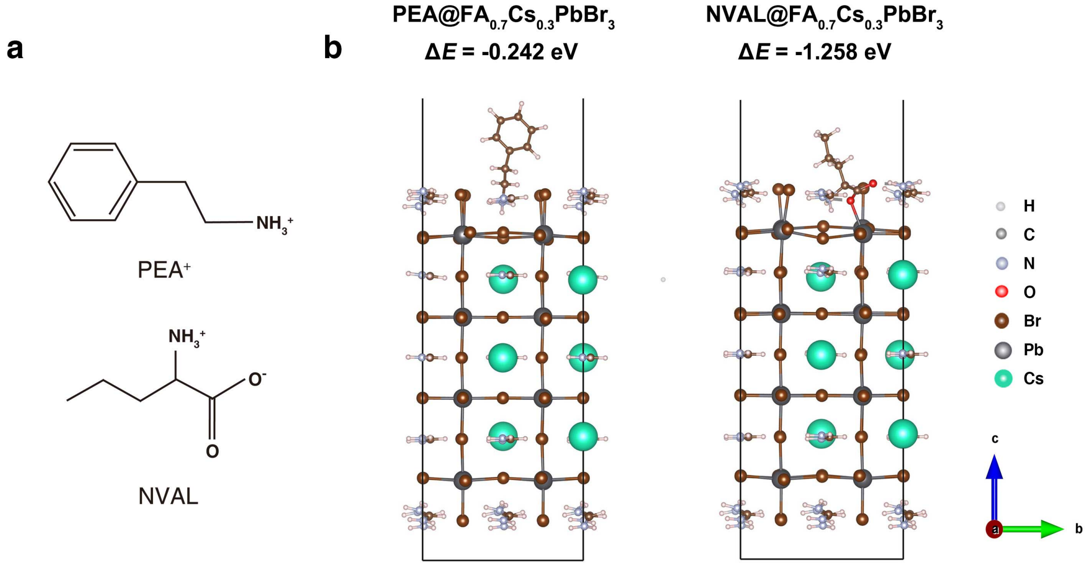

Supplementary Figure 7 | DFT simulation of quasi-2D perovskite intermediate phases, coordinated with PEA and NVAL, respectively. (a) Molecular structures of PEA and NVAL, respectively. **(b)** The optimized geometries and formation enthalpies of intermediate phases surface-anchored with NVAL (NVAL@$FA_{0.7}Cs_{0.3}PbBr_3$) and PEA (PEA@$FA_{0.7}Cs_{0.3}PbBr_3$), respectively.

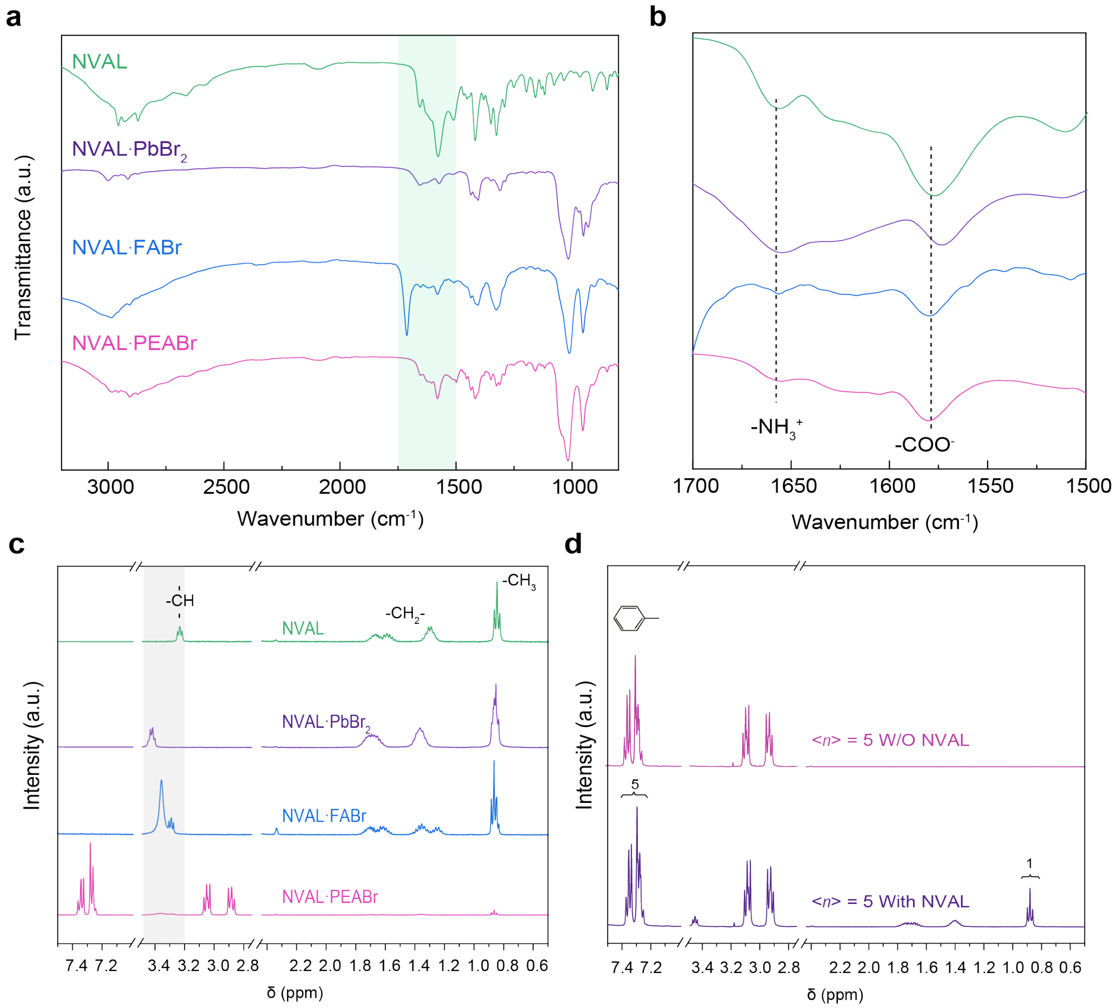

Supplementary Figure 8 | Characterizations of the coordination between NVAL and $[PbBr_6]^{4-}$ inorganic slabs. (a, b) FT-IR spectra of NVAL powder, and NVAL·$PbBr_2$, NVAL·FABr, NVAL·PEABr compounds (in DMSO solvent). The stretching vibration peaks of C=O and N-H bonds are located at 1578 and 1657 cm^{-1} in NVAL powder, while the C=O vibration red-shift to 1573 cm^{-1} in the DMSO·NVAL·$PbBr_2$ compound. **(c)** ^{1}H NMR spectra of NVAL and NVAL·$PbBr_2$, NVAL·FABr, NVAL·PEABr compounds in deuterated DMSO solution. Obvious downfield-shift of the tertiary methyl group proton resonance signals in NVAL after incorporation of $PbBr_2$ can be observed. **(d)** ^{1}H NMR spectra of NVAL and quais-2D perovskite with and without NVAL, the NMR data indicated that perovskite film keeps the initial PEABr : NVAL ratio well.

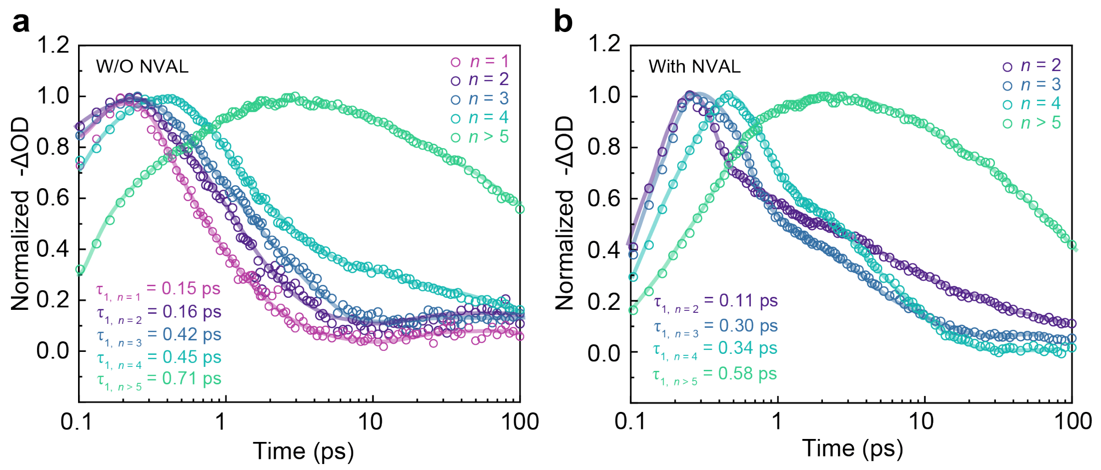

Supplementary Figure 9 | TA kinetics of the quasi-2D film without and with NVAL. TA traces as a function of decay time and extracted τ_1 of different phases for quasi-2D perovskite films **(a)** without and **(b)** with NVAL.

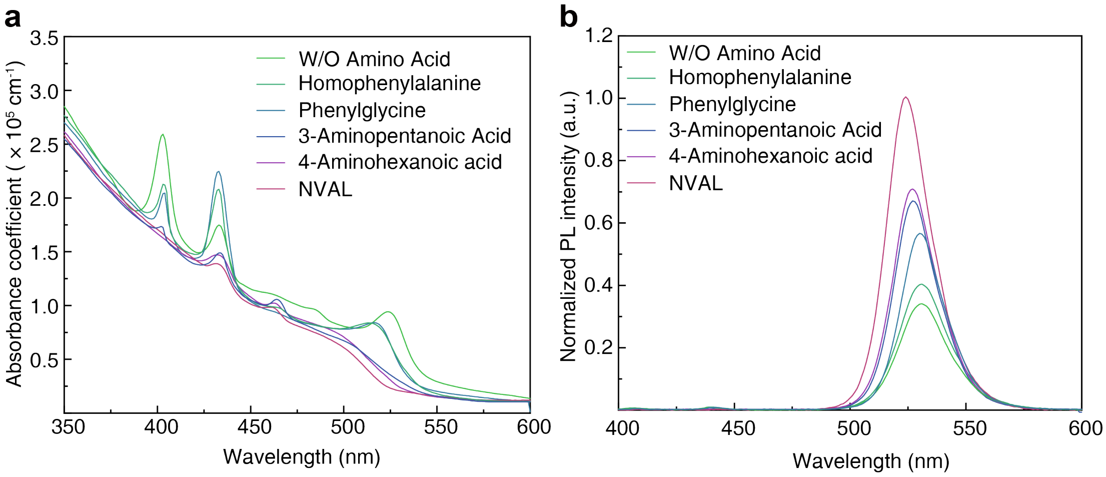

Supplementary Figure 10 | Spectroscopic measurements of quasi-2D film incorporated with different amino acids. (a) Steady-state UV-vis and **(b)** steady-state PL spectra of the quasi-2D films (<*n*> = 5).

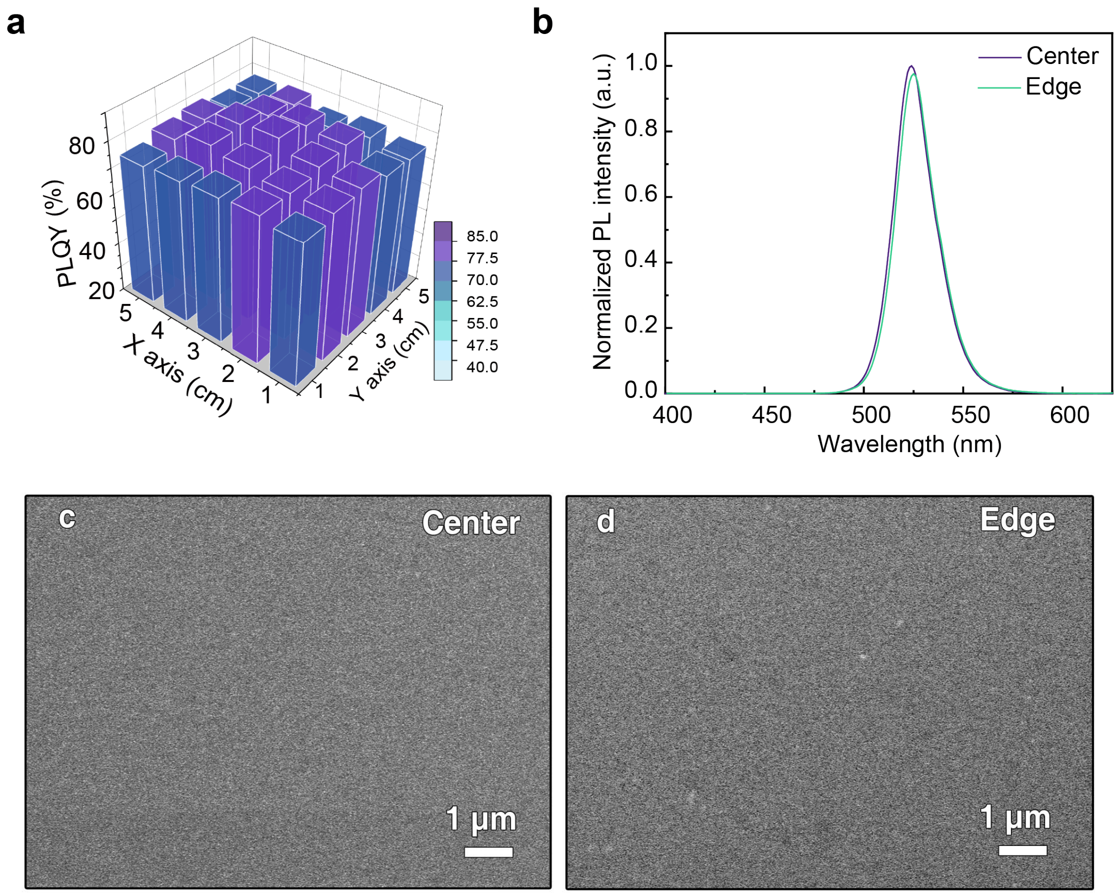

Supplementary Figure 11 | PL and SEM measurements of NVAL treated large-area quasi-2D film. **(a)** PLQY of the large-area quasi-2D film with film area up to 25 cm^2; PLQY is present using a 5 × 5-pixel fashion to distinguish the center- and edge-region (excitation intensity at ~ 10 nJ cm^{-2}). **(b)** PL spectra of the film at different regions. SEM images of the film at **(c)** center- and **(d)** edge-region.

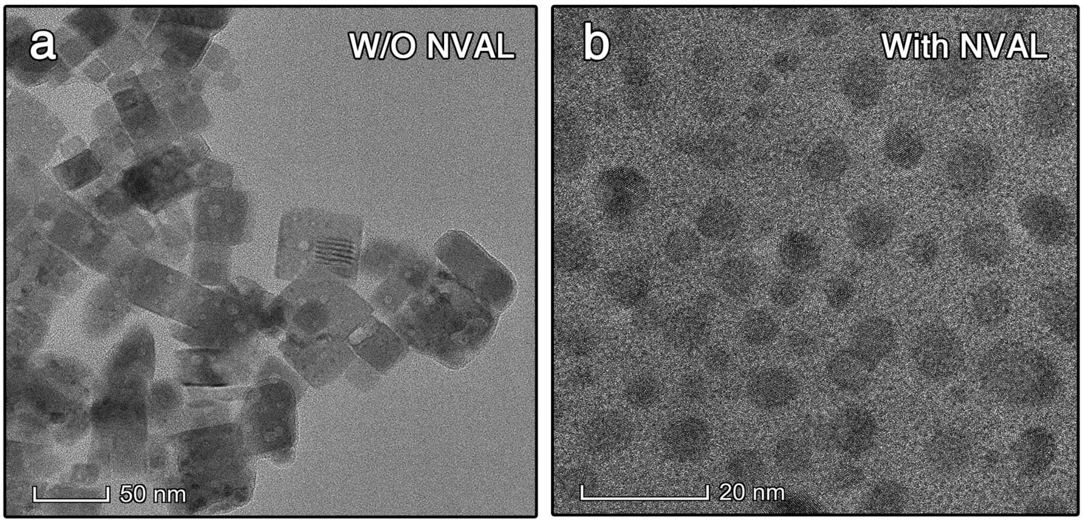

Supplementary Figure 12 | TEM measurement of quasi-2D films. TEM images of the film **(a)** without and **(b)** with NVAL (<*n*> = 5).

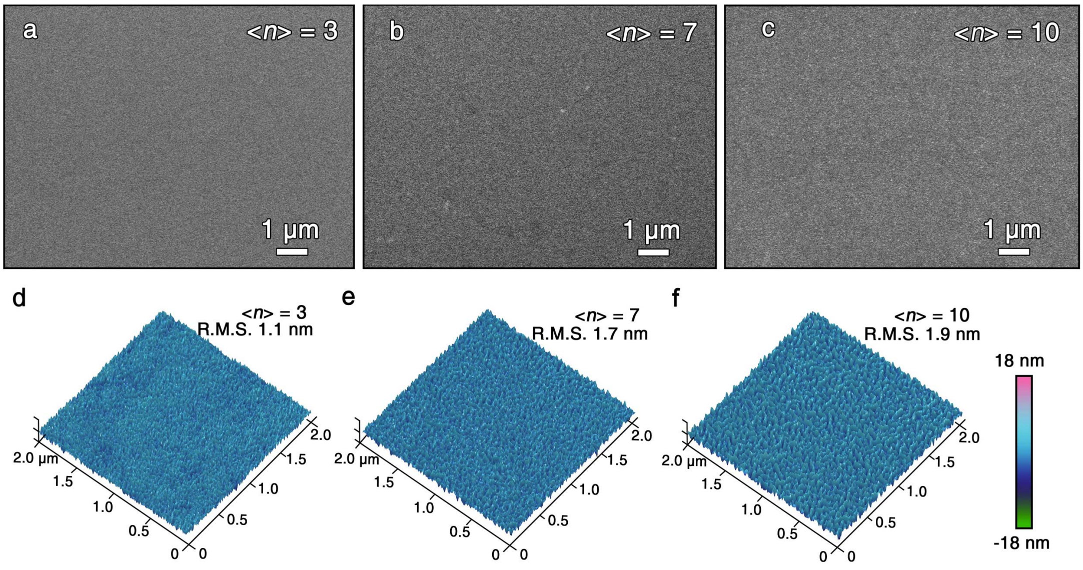

Supplementary Figure 13 | SEM and AFM measurements of NVAL treated quasi-2D films with different <*n*>-values on glass substrate. (a-c) SEM images of the quasi-2D films with <*n*> = 3, 7, and 10. **(d-f)** AFM images and the extracted root mean square roughness of the quasi-2D films with <*n*> = 3, 7, and 10.

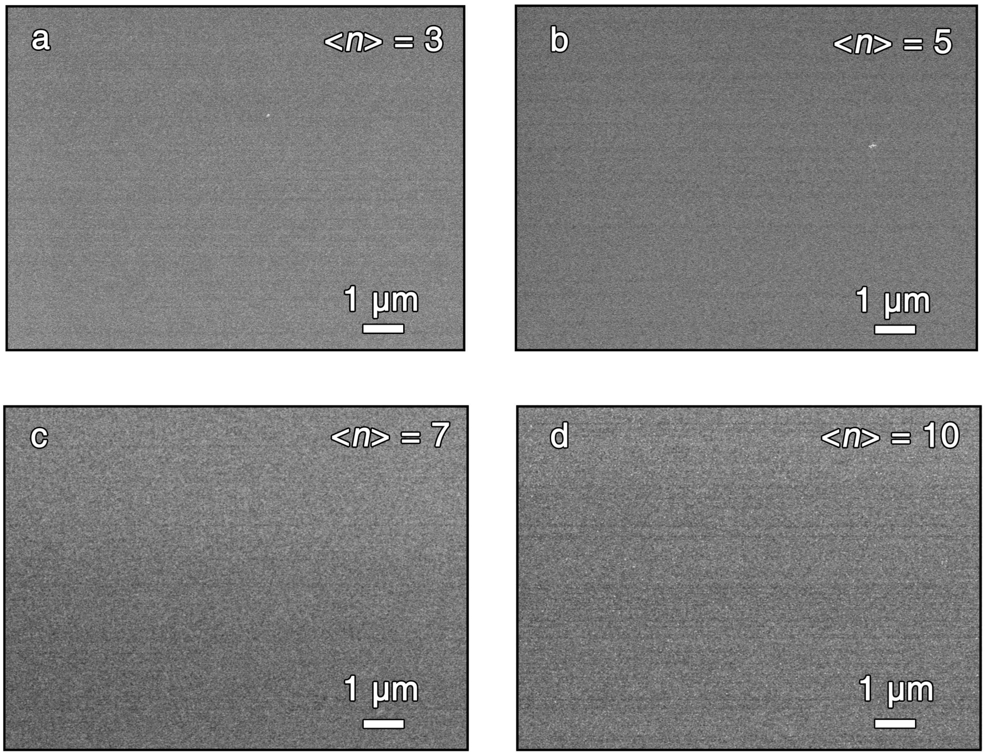

Supplementary Figure 14 | SEM measurements of NVAL treated quasi-2D films with different <*n*>-values on ITO/PEDOT:PSS/PFNBr substrates.

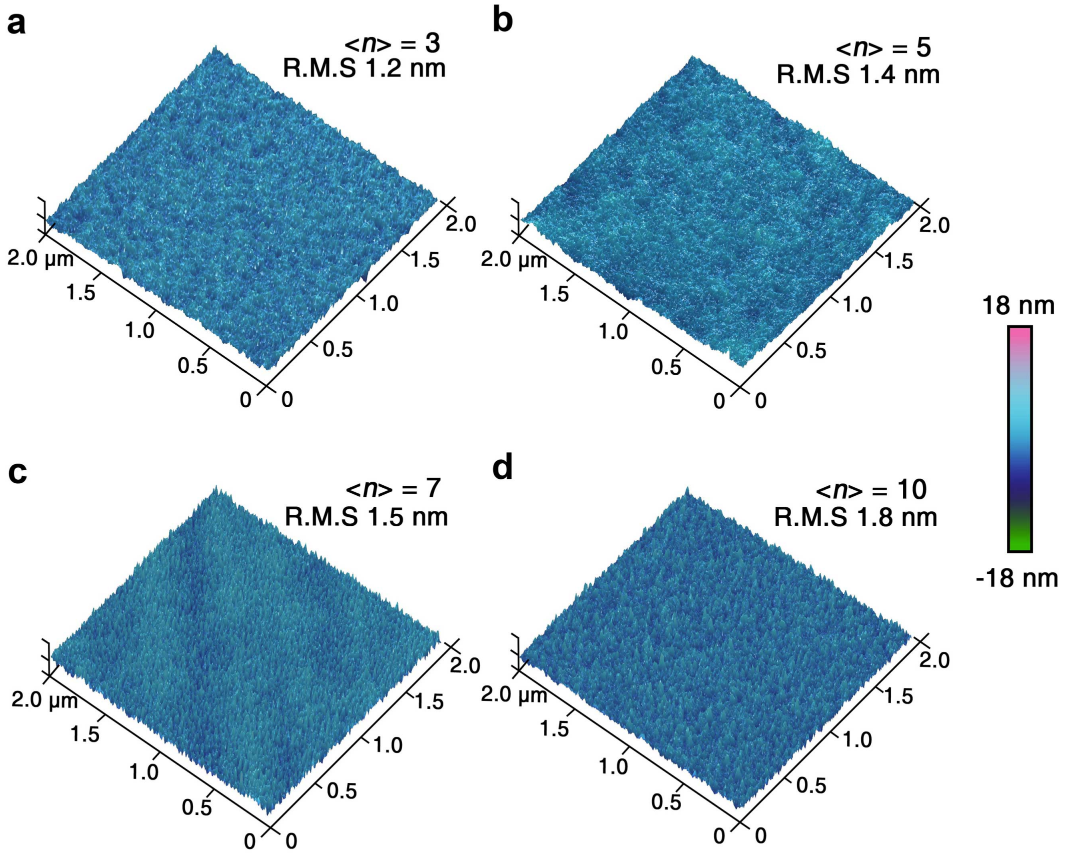

Supplementary Figure 15 | AFM measurements of NVAL treated quasi-2D films with different <*n*>-values on ITO/PEDOT:PSS/PFNBr substrates.

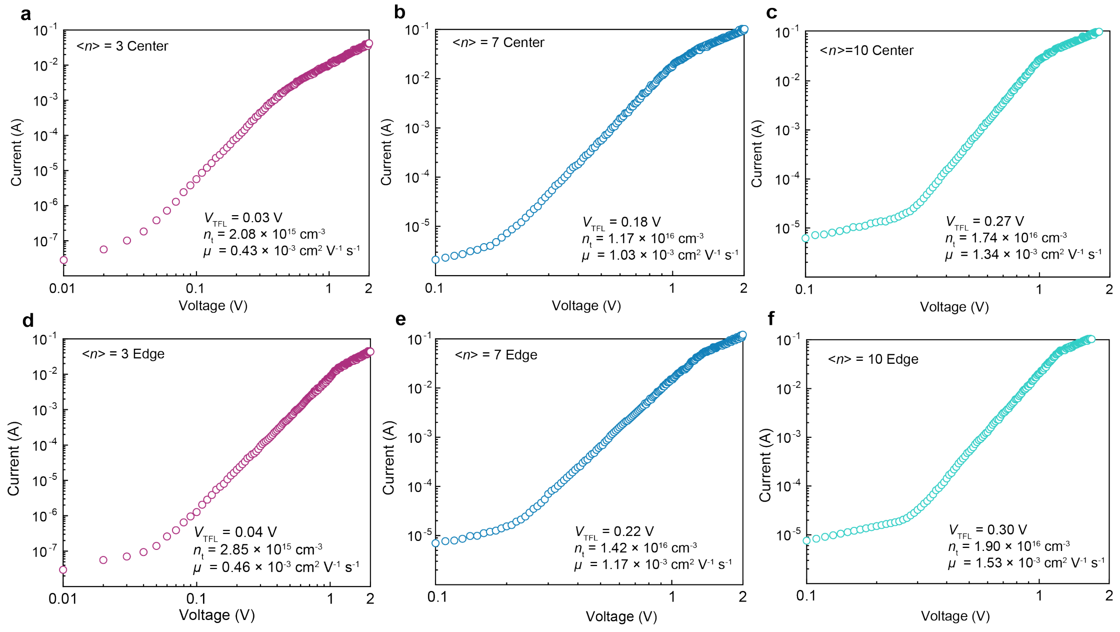

Supplementary Figure 16 | SCLC characterizations for quasi-2D perovskite films with different <*n*>-values at the center- and edge-region.

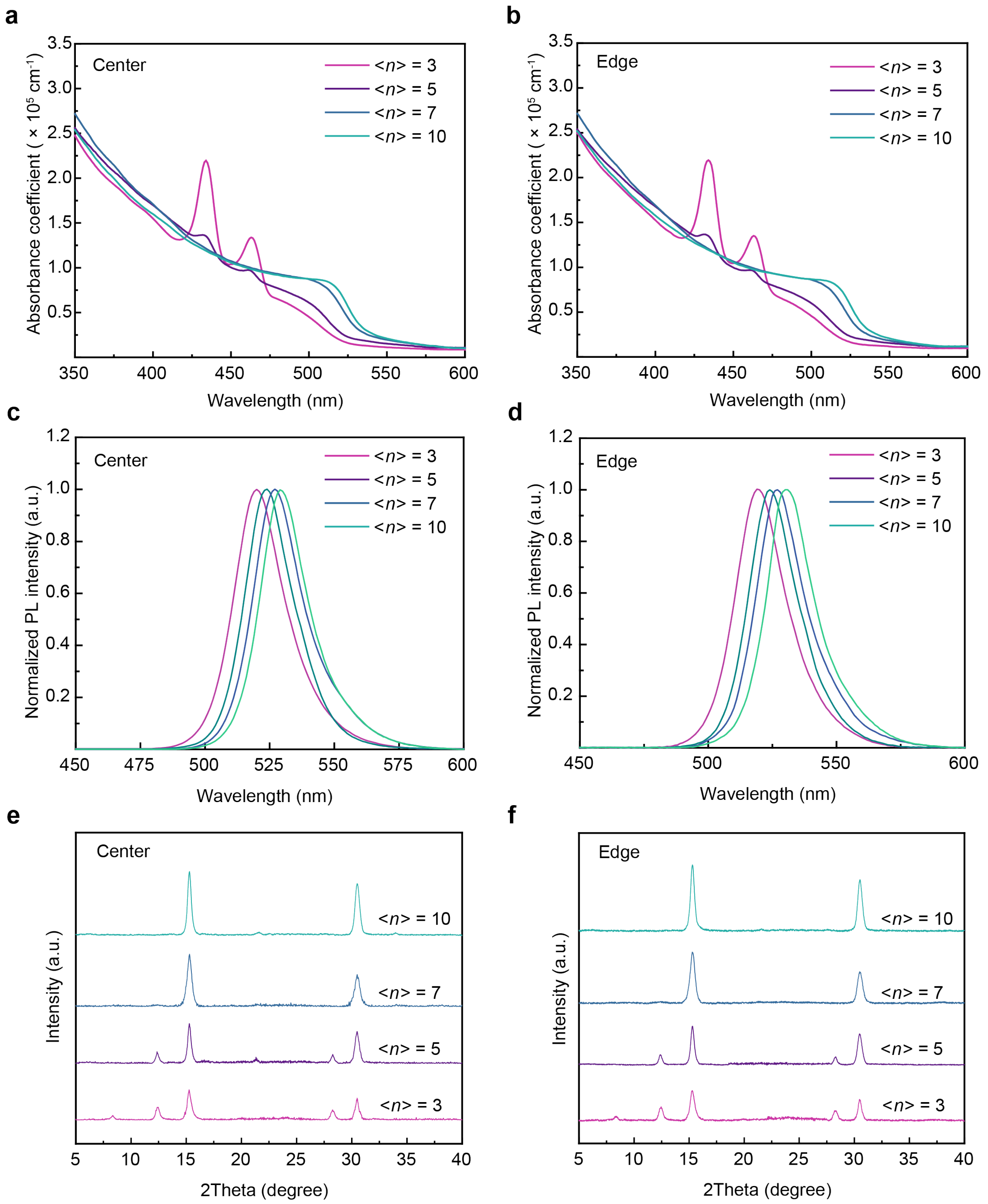

Supplementary Figure 17 | Spectroscopic measurements of NVAL treated quasi-2D films with different <*n*>-values. (a, b) steady-state PL spectra, **(c,d)** steady-state UV-vis spectra and **(e, f)** XRD patterns of the quasi-2D films in different regions.

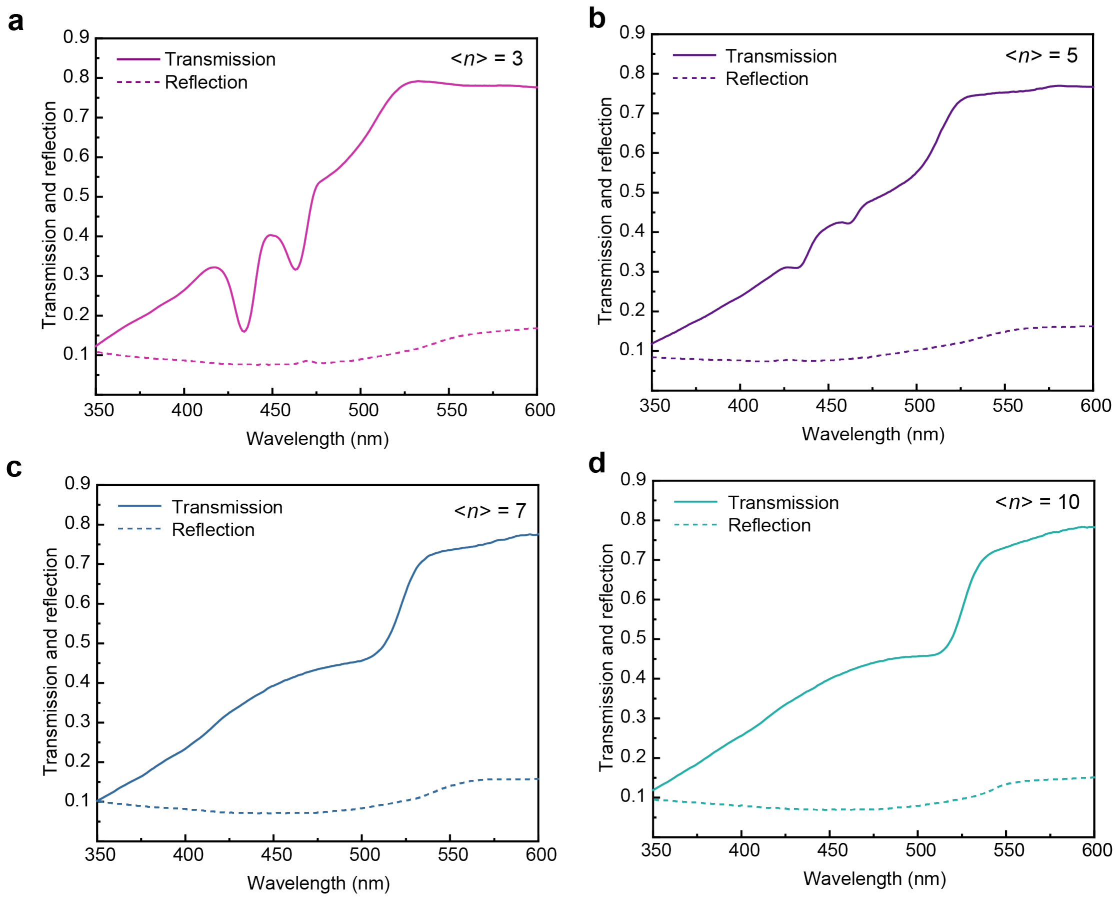

Supplementary Figure 18 | The transmission and reflection spectra of the quasi-2D perovskite films with different <*n*>-values. According to the transmission and reflection spectra, the corresponding absorption spectra can be calculated through the following equation[1]:

$$\alpha(\lambda) = -\frac{1}{l} ln \frac{T}{1-R} \tag{1}$$

where, α is absorption coefficient, T is transmittance, R is reflectance, and l is the average film thickness of layers.

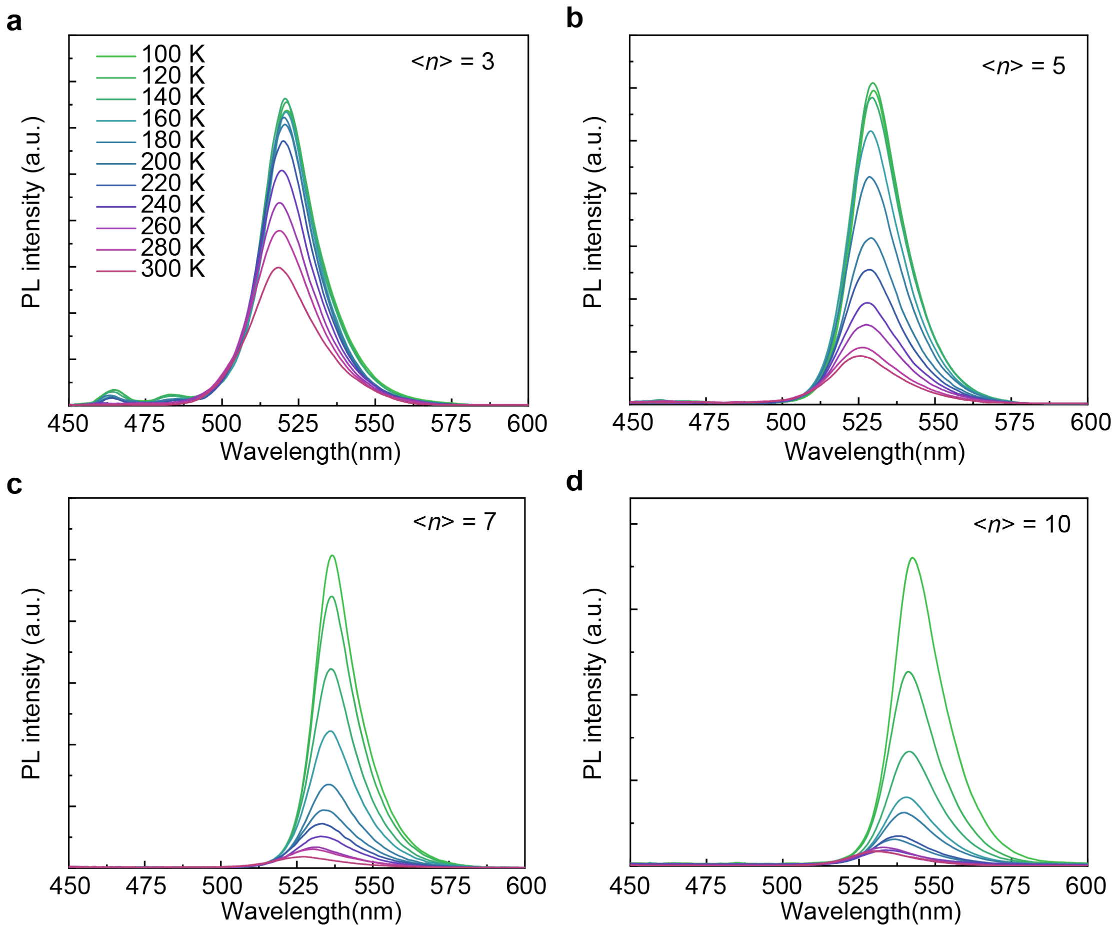

Supplementary Figure 19 | Temperature-dependent PL measurements of the NVAL treated quasi-2D films with different <*n*>-values. The exciton binding energies (E_b) of different films were extracted from the temperature-dependent PL intensity. According to previous reports, the temperature-dependent PL intensity curve fits well with the formula[2]:

$$I(T) = \frac{I_0}{1+Ae^{-E_b/k_B T}} \tag{2}$$

where, I_0, k_B and A are PL intensity at 0 K, Boltzmann constant, and constant that depends on the material itself, respectively.

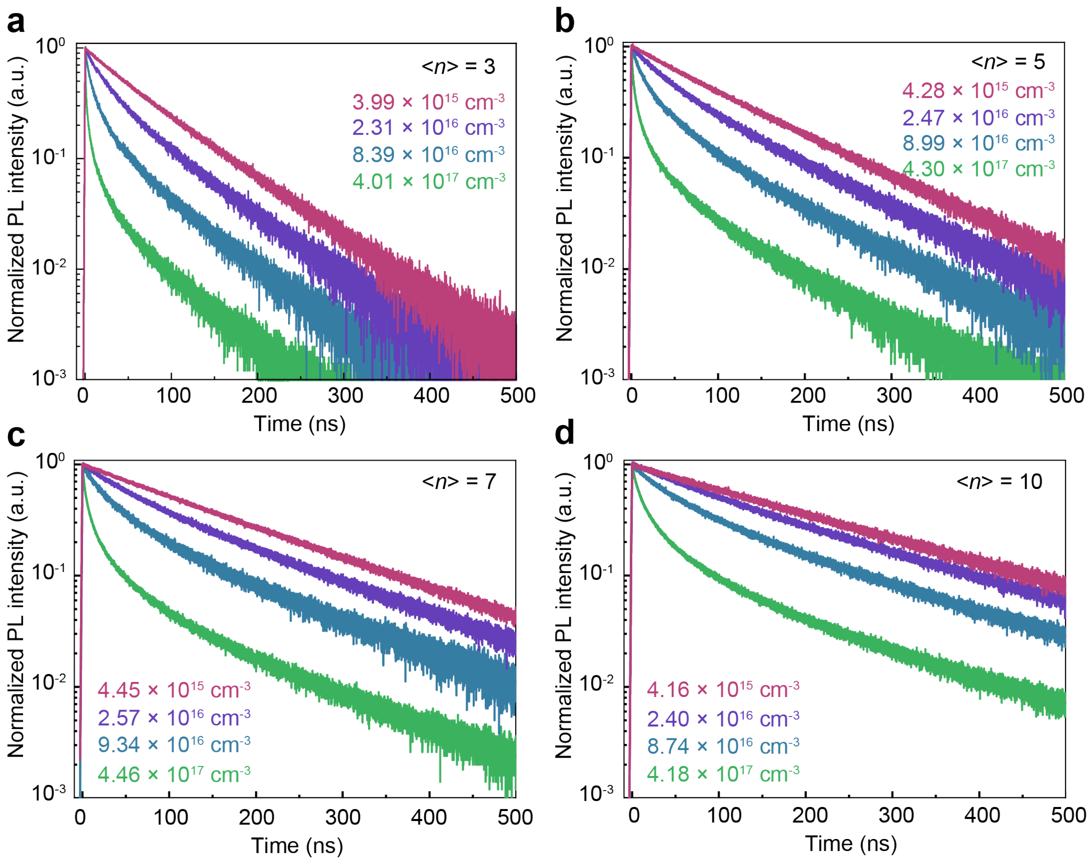

Supplementary Figure 20 | TRPL spectra for different carrier densities of NVAL treated quasi-2D films with different <*n*>-values. Under the low carrier densities, the PL intensity decays fit well with the mono-exponential relationship. The increased decay rate under high carrier densities indicates that the high-order recombination pathways gradually dominate.

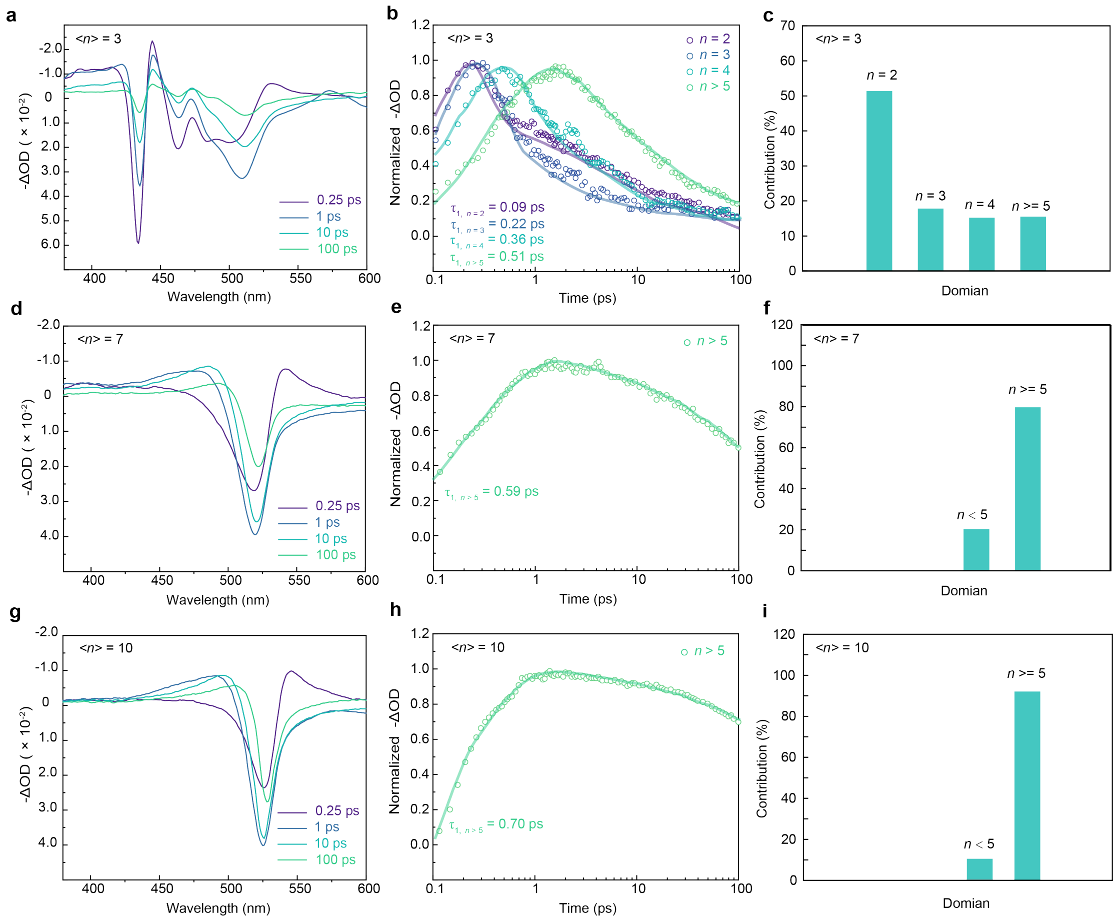

Supplementary Figure 21 | TA measurements for quasi-2D perovskite films with different <*n*> values at center-regions. (**a, d, g**) TA spectra at selected timescales; (**b, e, h**) TA spectra for different phases as a function of delay time; and (**c, f, i**) relative presence of different n domain according to the amplitude of GSBs in TA spectra at around 250 fs. The TA measurements for <*n*> = 5 film can be found in Fig. 2 and Supplementary Figure 10.

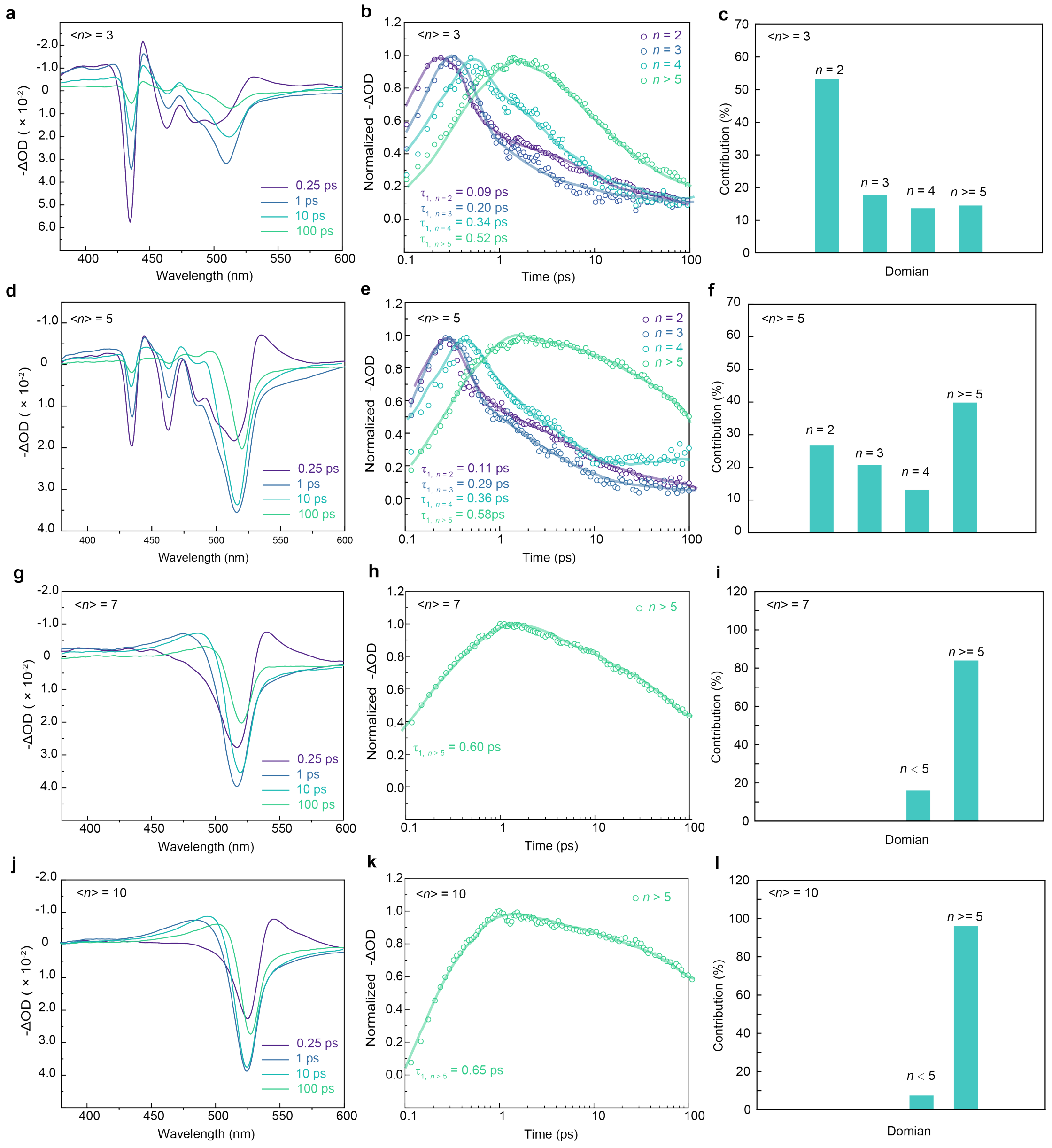

Supplementary Figure 22 | TA measurements for quasi-2D perovskite films with different <*n*> values at edge-regions. (**a, d, g, j**) TA spectra at selected timescales; (**b, e, h, k**) TA spectra for different phases as a function of delay time; and (**c, f, i, l**) relative presence of different n domain according to the amplitude of GSBs in TA spectra at around 250 fs.

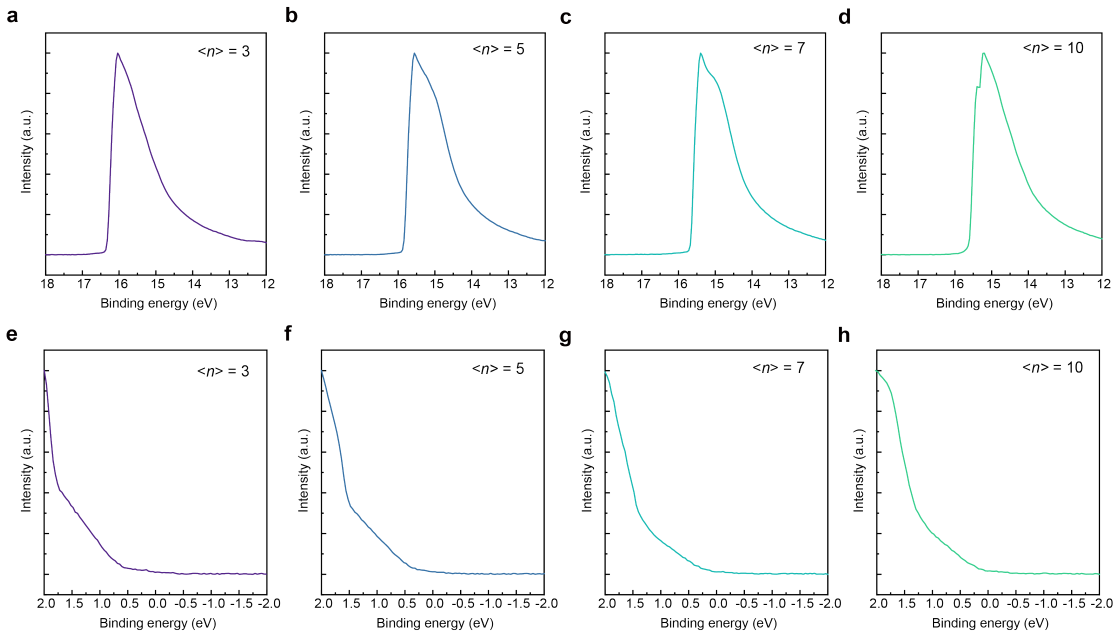

Supplementary Figure 23 | UPS spectra for quasi-2D perovskite films with different <*n*>-values. (**a-d**) UPS spectra close to the onset region for perovskite films. (**e-f**) Valence band spectra of perovskite films. The work function is calculated as Φ = 21.2 - onset, the VBM is determined by the linear extrapolation of the leading edge of the valence band to zero baseline intensity[3].

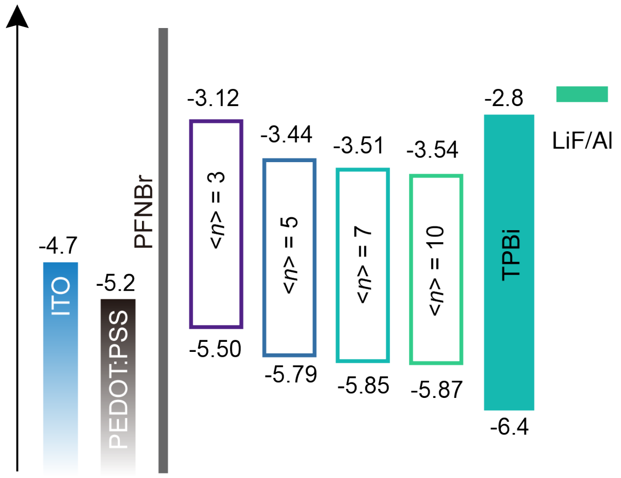

Supplementary Figure 24 | Band alignment of each function layer in the PeLED devices.

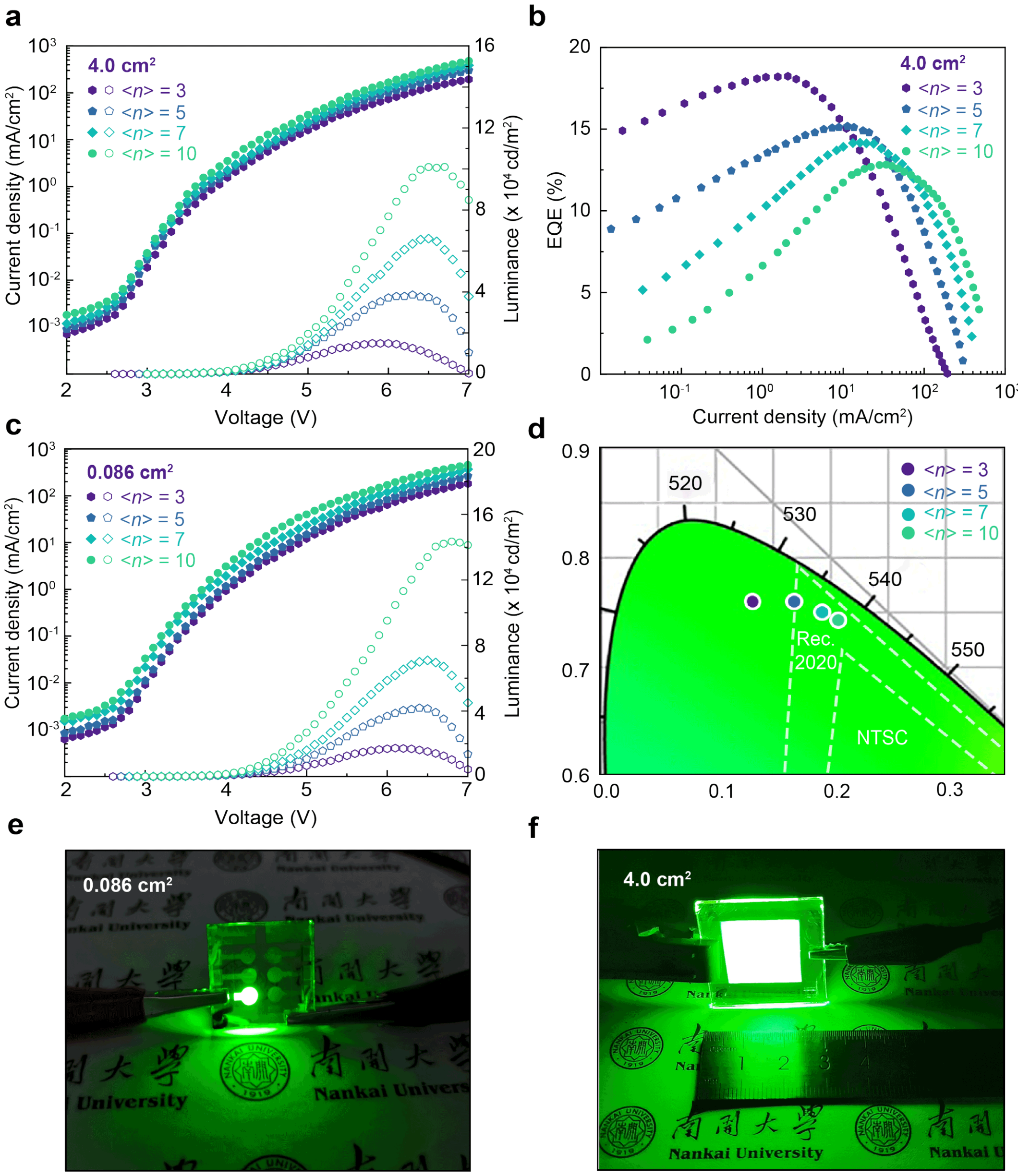

Supplementary Figure 25 | Device performance of the resulting NVAL treated quasi-2D PeLEDs. (a) *J-L-V* and **(b)** *EQE-J* characteristics of the PeLEDs with active areas of 4.0 cm^2. **(c)** *J-L-V* characteristics of the PeLEDs with active areas of 0.086 cm^2. **(d)** the CIE color coordinates for different <*n*>-value devices. Digital photos of the devices with active areas of **(e)** 0.086 and **(f)** 4.0 cm^2 operated under biased voltage of 4.5 V (<*n*> = 3).

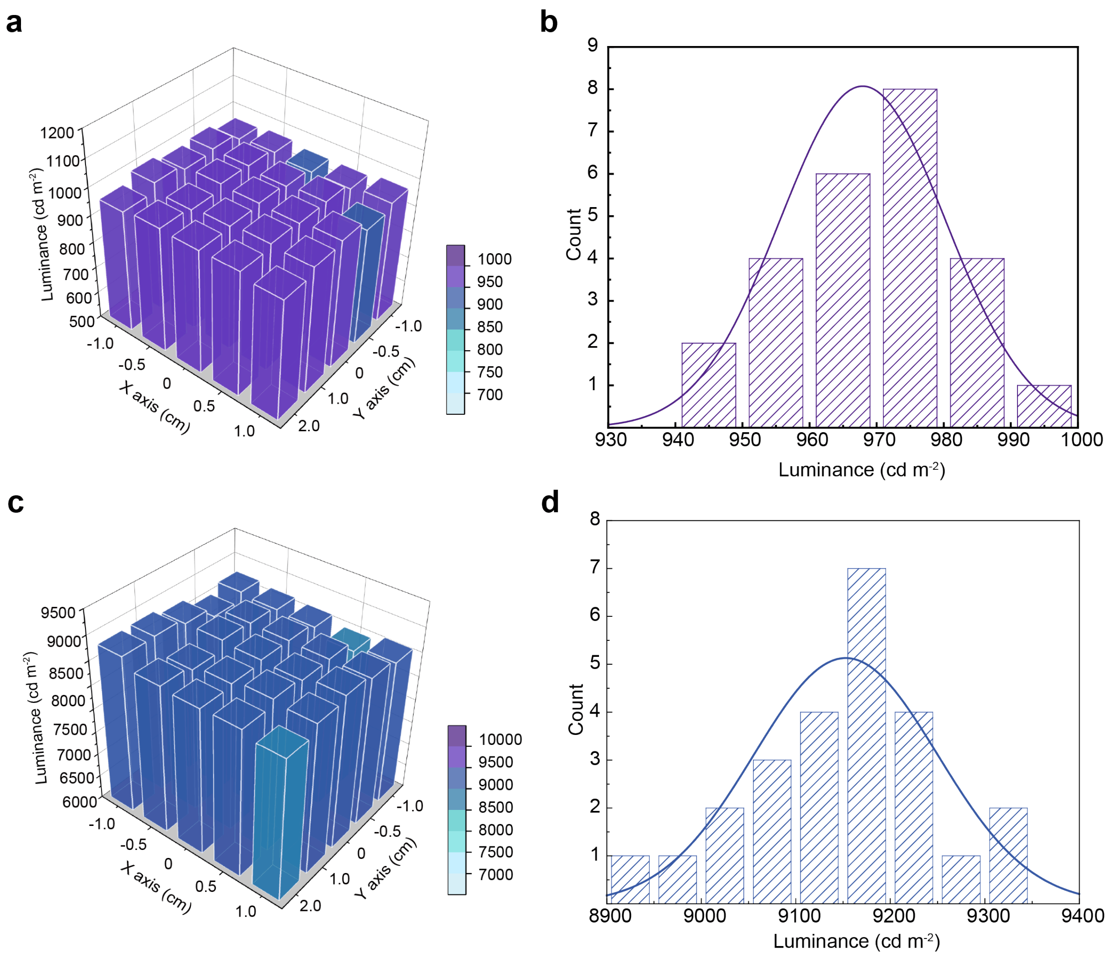

Supplementary Figure 26 | EL uniformity measurements. Luminance of the PeLED device (9.0 cm^2, <*n*> = 3) under biased voltage of **(a)** 4.0 V and **(b)** 5.0 V, luminance is present using a 5 × 5-pixel fashion to distinguish the center- and edge-region. Statistical luminance for the 25 pixels, under biased voltage of **(c)** 4.0 V and **(d)** 5.0 V.

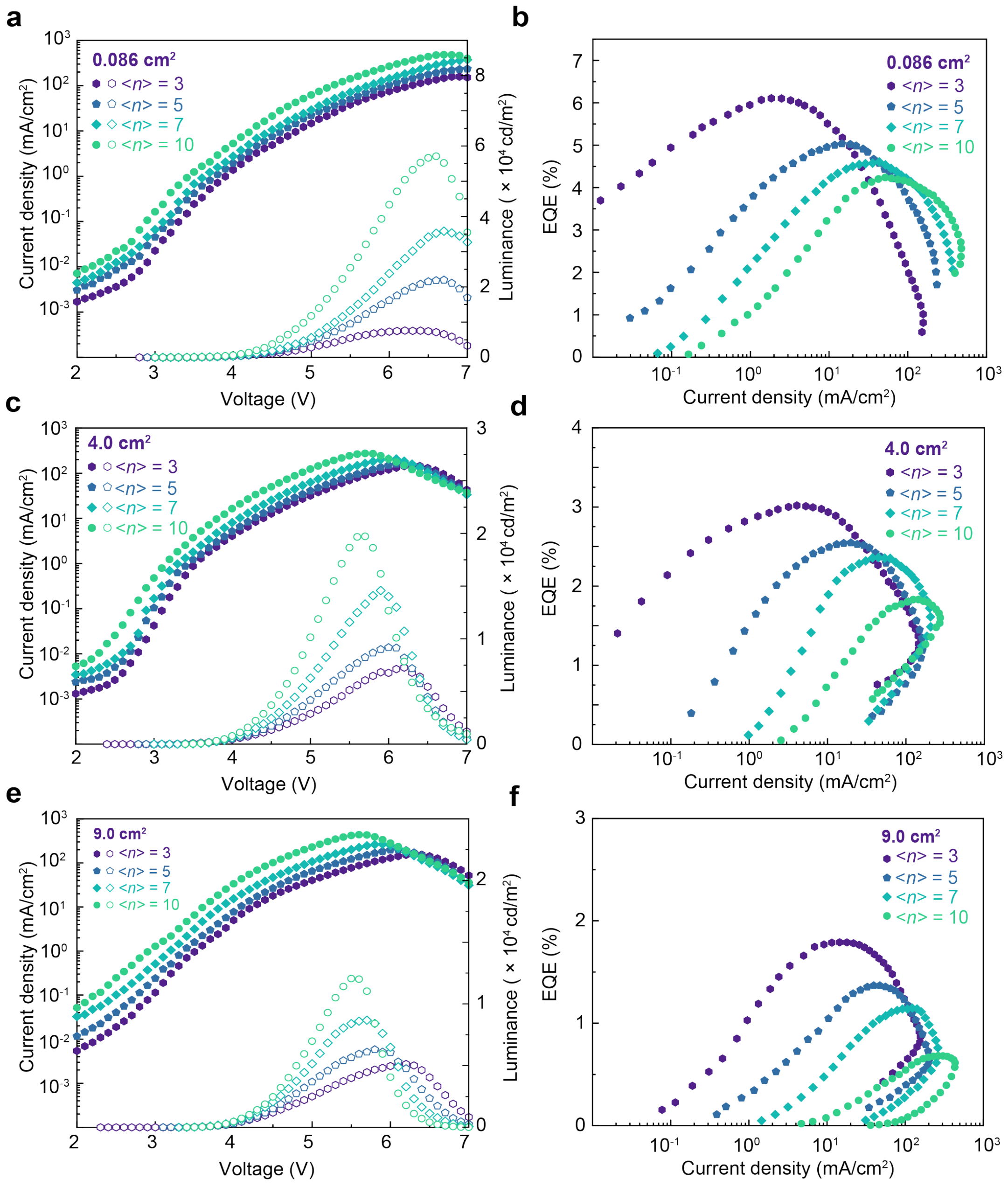

Supplementary Figure 27 | Device performance of the resulting quasi-2D PeLEDs without NVAL treated. *J-L-V* and *EQE-J* characteristics of the PeLEDs with active areas of **(a, b)** 0.086 cm^2, **(c, d)** 4.0 cm^2 and **(e, f)** 9.0 cm^2.

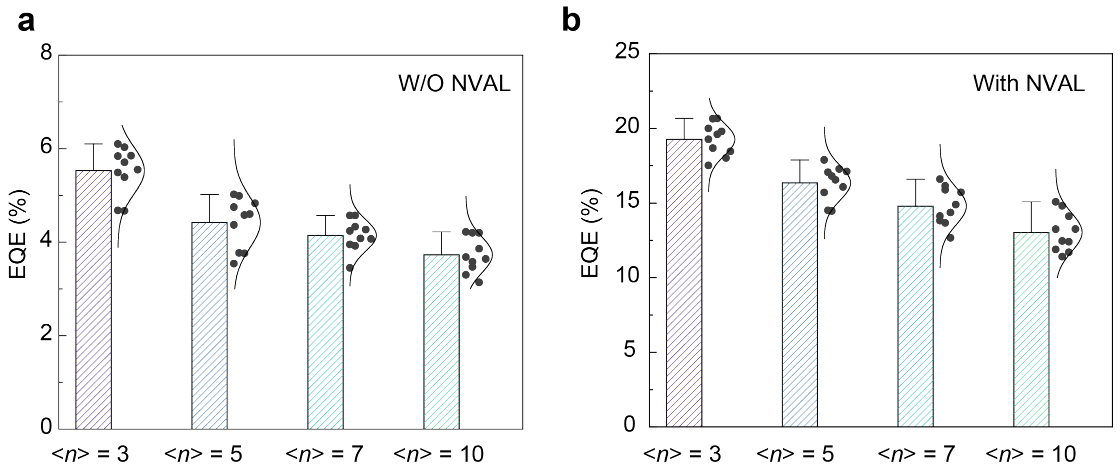

Supplementary Figure 28 | Histograms of peak EQE in the resulting PeLEDs. PeLEDs based on the quasi-2D films **(a)** without and **(b)** with NVAL (active area, 0.086 cm^2).

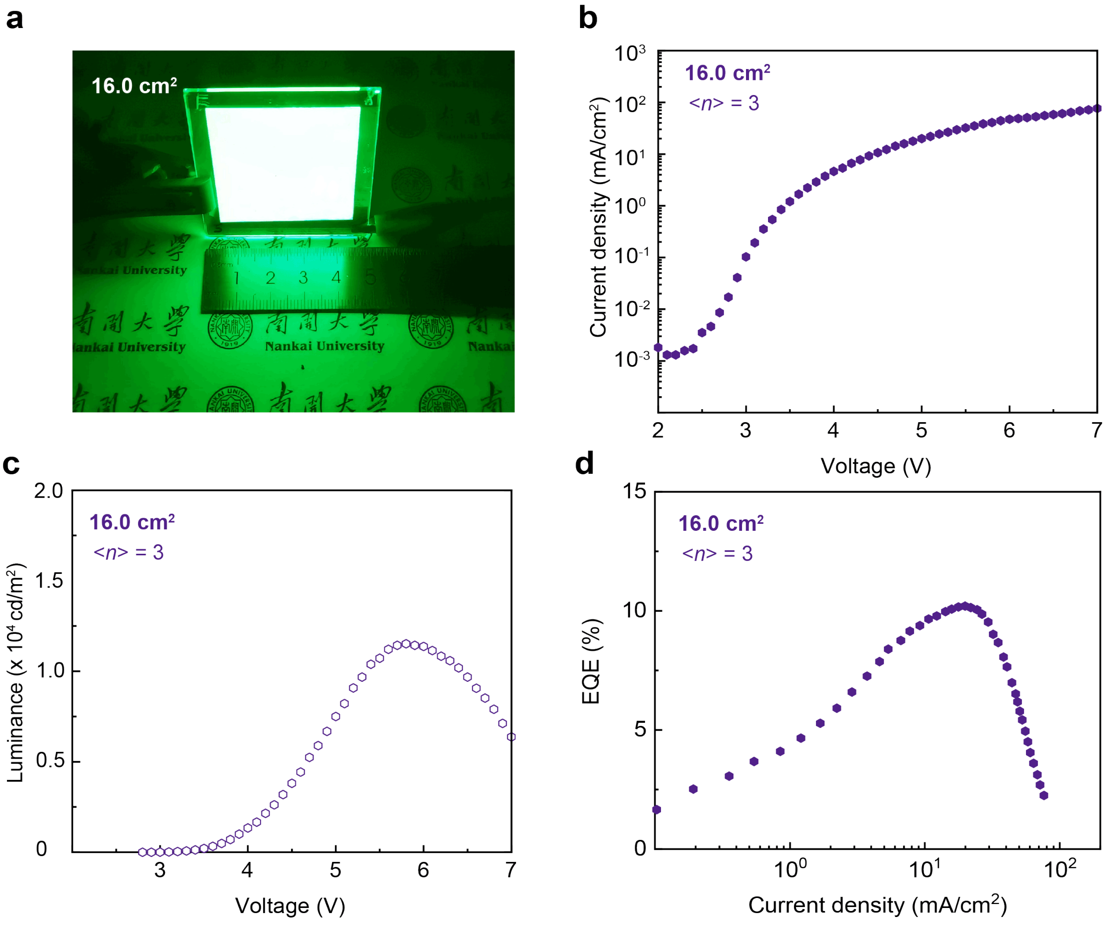

Supplementary Figure 29 | Device performance of the quasi-2D PeLEDs with active area of 16.0 cm^2. (a) Photographs of the device, **(b)** *J -V*, **(c)** *L-V*, **(d)** *EQE-J* characteristics of the device. We attribute the device performance degradation to the increased series resistance in the device.

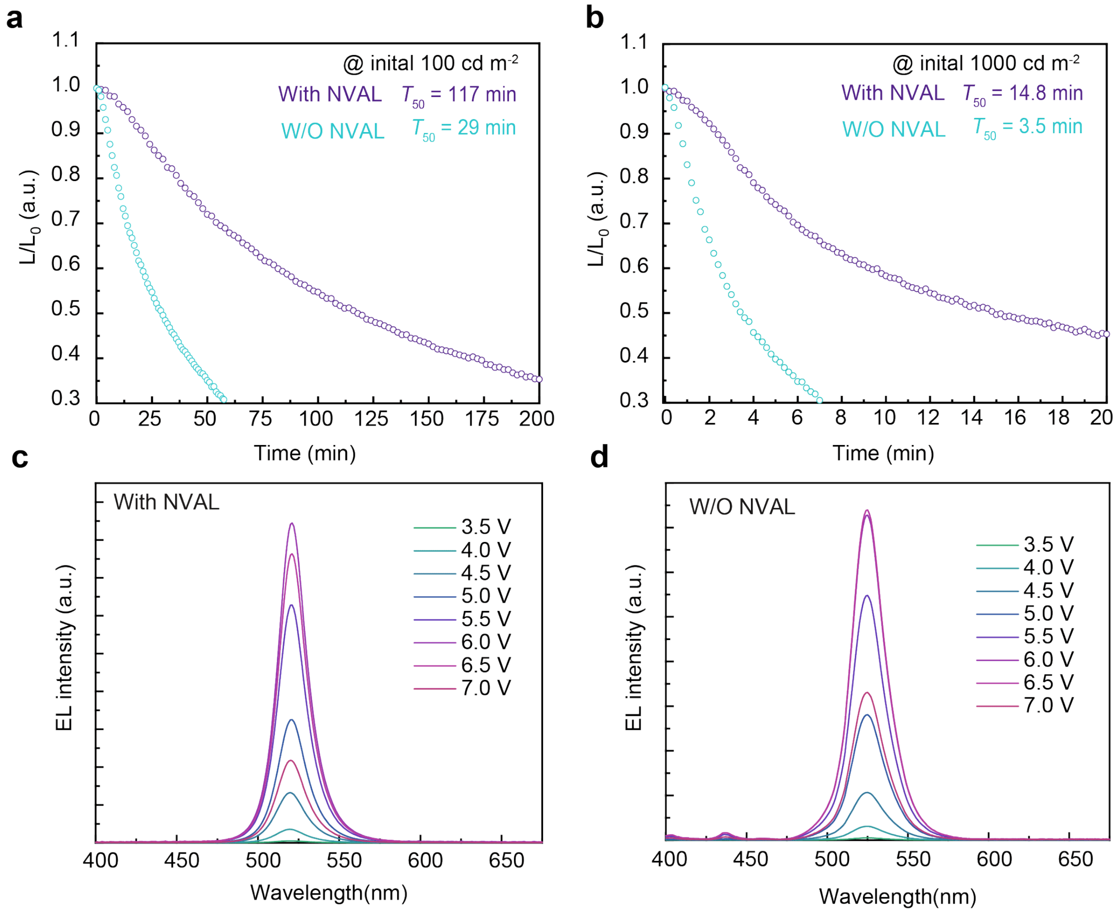

Supplementary Figure 30 | Stability measurements of the <*n*> = 3 quasi-2D PeLEDs. Lifetime measurement (T_{50}) of the device at an initial luminance of **(a)** 100 cd m^{-2} and **(b)** 1,000 cd m^{-2}. **(c, d)** EL spectra of the device at different voltage bias.

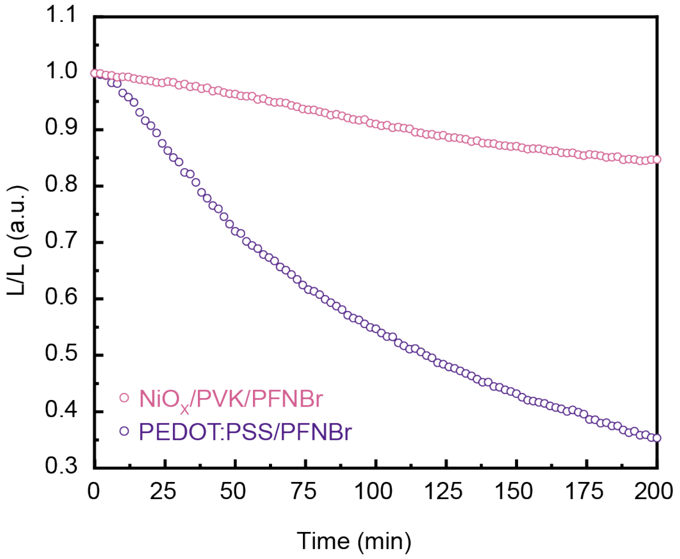

Supplementary Figure 31 | Lifetime measurement of the <*n*> = 3 quasi-2D PeLEDs based on NiO_X/PVK/PFNBr and PEDOT:PSS/PFNBr HTLs, respectively (initial luminance, 100 cd m^{-2}).

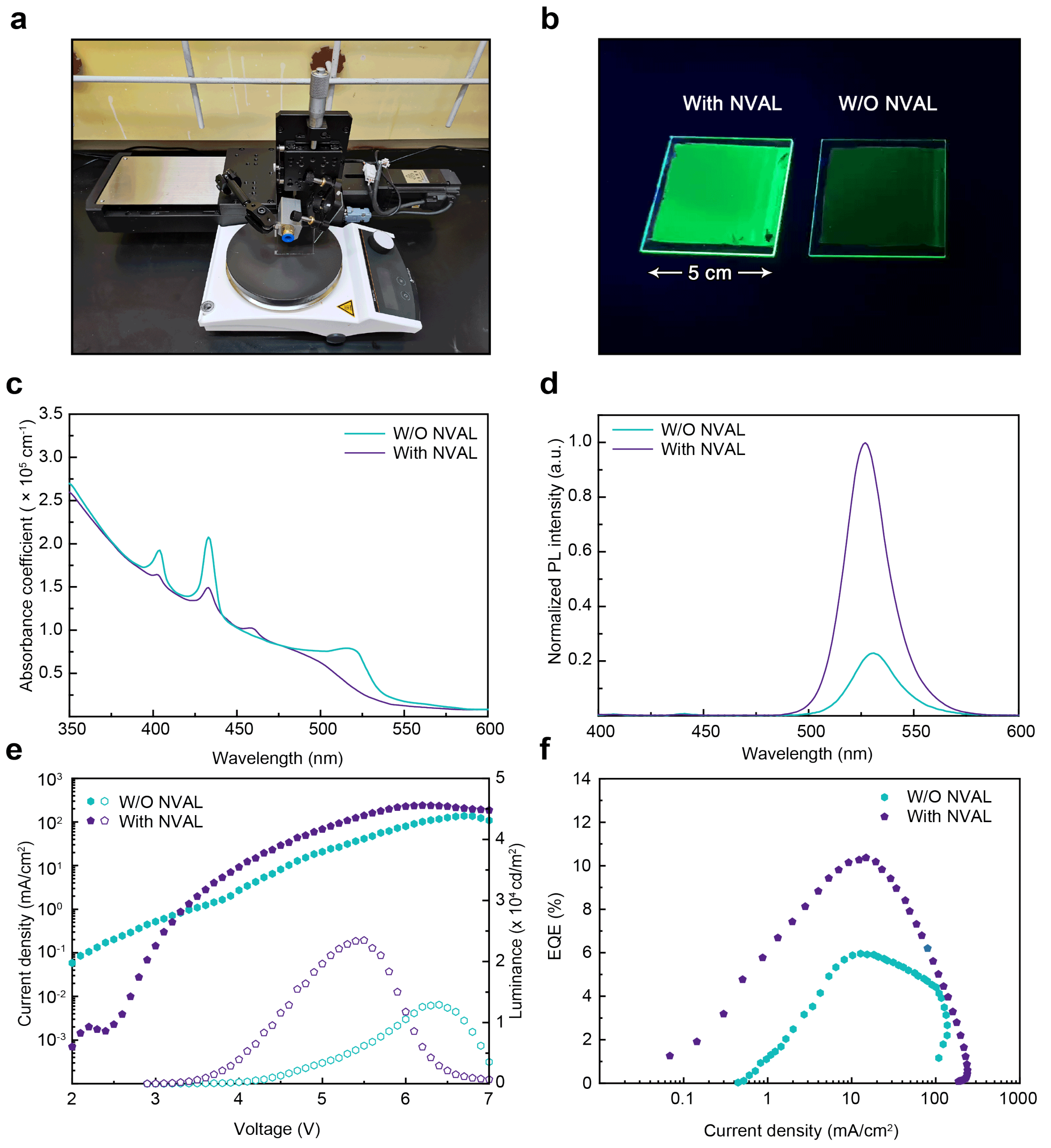

Supplementary Figure 32 | PeLEDs fabricated by the blade-coating technology. (a) Photographs of blade-coating equipment. **(b)** PL images **(c)** Steady-state UV-vis and **(d)** steady-state PL spectra of the large-area perovskite films. **(e)** Current density-luminance-voltage (*J-L-V*) and (f) EQE-current density (*EQE-J*) characteristics of PeLEDs, with an active area of 1.0 cm^2, $<n>$ = 5. Based on the NVAL-doped film, the prepared PeLED showed an efficiency of 10.2% with an active area of 1.0 × 1.0 cm^2, which is much higher than that of the undoped device. The NVAL modulation strategy is therefore also compatible with the blade-coating technology.

Supplementary Table 1 | Molecular structures of different amino acids; films' average PLQY and devices' average EQE of different amino acid-containing systems (<*n*> = 5).

Amino acids	NVAL	4-Aminohexanoic acid	3-Aminopentanoic Acid	Phenylglycine	Homo-phenylalanine
Molecule Structure	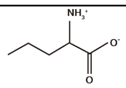	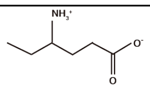	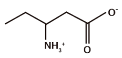	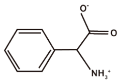	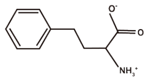
Average PLQY	80.3 %	57.1 %	54.4 %	43.5 %	38.7 %
Average EQE (0.086 cm^2)	16.4 %	13.0 %	10.7 %	8.2 %	6.4 %

Supplementary Table 2 | Summary of calculated k_{trap}, $k_{exciton}$, k_1, k_2 and k_3 for NVAL treated quasi-2D films with different <*n*>-values.

<*n*>-value	K_{trap} (s^{-1})	$K_{exciton}$ (s^{-1})	k_1 (s^{-1})	k_2 ($cm^3\ s^{-1}$)	k_3 ($cm^6\ s^{-1}$)
3	$1.9(\pm0.2) \times 10^6$	$1.0(\pm0.5) \times 10^7$	$1.2(\pm0.7) \times 10^7$	$1.1(\pm0.2) \times 10^{-9}$	$8.2(\pm1.4) \times 10^{-27}$
5	$2.2(\pm0.1) \times 10^6$	$6.1(\pm0.4) \times 10^6$	$8.3(\pm0.5) \times 10^6$	$4.6(\pm0.4) \times 10^{-10}$	$2.1(\pm1.0) \times 10^{-27}$
7	$2.4(\pm0.1) \times 10^6$	$3.4(\pm0.6) \times 10^6$	$5.9(\pm0.7) \times 10^6$	$2.4(\pm0.3) \times 10^{-10}$	$6.7(\pm0.7) \times 10^{-28}$
10	$2.6(\pm0.2) \times 10^6$	$2.2(\pm0.4) \times 10^6$	$4.8(\pm0.6) \times 10^6$	$1.3(\pm0.4) \times 10^{-10}$	$2.9(\pm0.9) \times 10^{-28}$

Supplementary Table 3 | Summary of the device performance for NVAL treated PeLEDs with active area of 9.0 cm^2.

<*n*>-value	Max. EQE (%)	Ave. EQE (%)	Max. Lum. (cd m^{-2})	EL Wavelength (nm)	FWHM (nm)	Current density @ Max. EQE (mA cm^{-2})	Current density @ Max. Lum. (mA cm^{-2})
3	16.4	14.2	17,260	520	21	1.0	89.1
5	13.2	11.7	30,110	527	22	9.2	149.7
7	11.2	9.4	54,270	531	23	19.6	245.2
10	9.5	7.9	91,650	532	24	41.0	374.5

Supplementary Table 4 | Comparison of device performance for the state-of-the-art large-area PeLEDs reported to date.

Type of Perovskites	Wavelength (nm)	Active area (cm^2)	Peak EQE (%)	References
Nanocrystal	530	3.0	2.0	Ref.4
Nanocrystal	512	4.0	~ 11	Ref.5
Nanocrystal	531	4.0	16.3	Ref.6
3D	799	1.2	5.7	Ref.7
3D	800	9.0	12.1	Ref.8
film from blade-coating	730	1.0	12.7	Ref.9
**Quasi-2D**	**520**	**9.0**	**16.4**	**This work**

Supplementary Table 5 | Summary of the device performances for NVAL treated PeLEDs with active area of 0.086 cm^2.

<*n*>-value	Max. EQE (%)	Ave. EQE (%)	Max. Lum. (cd m^{-2})	EL Wavelength (nm)	FWHM (nm)	Current density @ Max. EQE (mA cm^{-2})	Current density @ Max. Lum. (mA cm^{-2})
3	21.4	19.3	17,320	520	21	0.23	74.0
5	17.9	16.4	41,820	527	22	5.2	144.9
7	16.6	14.8	70,900	530	23	13.0	216.9
10	15.1	13.1	143,410	532	23	27.1	396.4

Supplementary Table 6 | Comparison of device performance for the state-of-the-art small-area PeLEDs reported to date.

Type of Perovskites	Wavelength (nm)	Peak EQE (%)	Max. Lum. (cd m^{-2})	T_{50}	References
3D	525	20.3	14,000	10.42 min @ 7,130 cd m^{-2}	**Ref.** 10
3D	520	16.2	50,270	6.2 min @ 10,000 cd m^{-2}	**Ref.** 11
3D	518	17.0	35,700	-	**Ref.** 13
3D	530	17.5	~38,000	-	**Ref.** 13
3D	795	20.1	-	46h @ 0.1 mA cm^{-2}	**Ref.** 14
3D	800	21.6	-	~20 h @ 60 mA cm^{-2}	**Ref.** 15
2D/3D	518	19.1	~50,000	-	**Ref.** 16
Nanocrystal	653	21.3	~500	~5 min @ 1.25 mA cm^{-2}	**Ref.** 17
Nanocrystal	516	18.7	~21,000	~30 min @1000cd	**Ref.** 18
Quasi-2D	514	15.5	19,540	~60 min @ 2 mA cm^{-2}	**Ref.** 19
Quasi-2D	532	14.4	9,120	~75 min @ 0.3 mA cm^{-2}	**Ref.** 20
**Quasi-2D**	**520**	**21.4**	**17,320**	**117 min @100cd**	**This work**

Supplementary Table 7 | Precursor compositions of quasi-2D perovskite film with different <*n*> values.

<*n*>-value	PEABr (mmol mL^{-1})	FABr (mmol mL^{-1})	CsBr (mmol mL^{-1})	$PbBr_2$ (mmol mL^{-1})	NVAL (mmol mL^{-1})	Bu_4PBF_4 (mmol mL^{-1})
3	0.26	0.18	0.08	0.4	0.032	0.012
5	0.16	0.224	0.096	0.4	0.032	0.012
7	0.114	0.24	0.103	0.4	0.032	0.012
10	0.08	0.252	0.108	0.4	0.032	0.012

Supplementary Note 1 | SCLC measurements

Trap state densities were obtained from the widely reported spatial charge-limited current (SCLC) model with an ITO/PEDOT:PSS/perovskites/MoO_3/Au structure[21]. Typically, the corresponding *I-V* responses can be divided into Ohmic, trap-filling and Child stages. In the low voltage stage (ohmic region), a linear increase in current with voltage was observed, which represented the trap state filling process. In the high voltage stage, the current showed a rapid nonlinear growth with voltage increased. The starting point of the second stage means the limitation of trap filling, where the voltage in this point (V_{TFL}) is proportional to the trap density, and the formula can be expressed as:

$$V_{TFL} = \frac{en_t l^2}{2\varepsilon\varepsilon_0} \quad (3)$$

where n_t is the trap density of the film, e is the elementary charge, ε_0 are the vacuum dielectric constant, ε and l are relative dielectric constant and thickness of the perovskite film.

The hole mobility (μ) can be then extracted from the child region according to the equation of:

$$I = \frac{9\varepsilon\varepsilon_0 \mu S V^2}{8l^3} \quad (4)$$

where I is the device current, S is the electrode area.

Supplementary Note 2 | DFT simulation

Density functional theory (DFT) calculations were performed by Vienna Ab initio Simulation Package (VASP) to obtain the formation enthalpies of perovskite intermediate phases coordinated with PEA (PEA@$FA_{0.7}Cs_{0.3}PbBr_3$) and NVAL (NVAL@$FA_{0.7}Cs_{0.3}PbBr_3$)[22]. The original $FA_{0.7}Cs_{0.3}PbBr_3$ surface (001) with FABr termination was created by a 2 × 2 × 4 cubic supercell containing 186 atoms. In NVAL@$FA_{0.7}Cs_{0.3}PbBr_3$ system, the amidogen of NVAL occupies the FA vacancy while the carboxyl fills the bromine vacancy and the oxygen of carboxyl group coordinated with Pb of $FA_{0.7}Cs_{0.3}PbBr_3$. In PEA@$FA_{0.7}Cs_{0.3}PbBr_3$ system, the PEA substitutes the FA vacancy in the surface of $FA_{0.7}Cs_{0.3}PbBr_3$. To screen off the spurious interactions, a vacuum of 25 Å was added along the *z*-direction. A uniform 3 × 3 × 1 Monkhorst-Pack k-point mesh and a plane-wave energy cutoff of 400 eV were adopted for the geometry optimization[23]. The Perdew-Burke-Ernzerhof (PBE) functional and projector-augmented wave (PAW) method were used to describe electron exchange-correlation interactions and electron-ion interaction[24,25]. The van der Waals interactions are described by the Grimme DFT-D3 approach[26]. The formation enthalpies of NVAL@$FA_{0.7}Cs_{0.3}PbBr_3$ and PEA@$FA_{0.7}Cs_{0.3}PbBr_3$ were calculated according to the following equations:

$$\Delta E(NVAL@FA_{0.7}Cs_{0.3}PbBr_3) = E_{NVAL@FA_{0.7}Cs_{0.3}PbBr_3} + E_{FA} - E_{FABr-surface} - E_{NVAL} \quad (5)$$

$$\Delta E(PEA@FA_{0.7}Cs_{0.3}PbBr_3) = E_{PEA@FA_{0.7}Cs_{0.3}PbBr_3} + E_{FA} - E_{FABr-surface} - E_{PEA} \quad (6)$$

Here, $E_{NVAL@FA_{0.7}Cs_{0.3}PbBr_3}$, $E_{PEA@FA_{0.7}Cs_{0.3}PbBr_3}$, E_{FABr}, E_{FA}, E_{NVAL}, E_{PEA} are the energies of NVAL@$FA_{0.7}Cs_{0.3}PbBr_3$, PEA@$FA_{0.7}Cs_{0.3}PbBr_3$ systems, FABr, FA, NVAL and PEA, $E_{FABr-surface}$ is the energy of $FA_{0.7}Cs_{0.3}PbBr_3$ (001) surface with FABr termination, $E_{V_{FA}@FABr-surface}$ is the energy of FABr terminated $FA_{0.7}Cs_{0.3}PbBr_3$ (001) surface containing an FA vacancy.

Supplementary Note 3 | Recombination kinetics analysis

Initial carrier density (N_0) calculation

In the experiment, the observed transmission change of probe pulse $x(t)$ is proportional to the photogenerated charge-carrier density $N(t)$ and can be formulated as:

$$N(t) = \varphi C x(t) \quad (7)$$

where φ refer to the photon-to-free-carrier conversion ratio, and C refers to the proportionality factor between the initial transmission change $x(0)$ and the photogenerated charge-carrier density N_0 described as follows:

$$N_0 = \frac{E\lambda\alpha(\lambda)}{hcA_{eff}}(1 - R_{pump}) \quad (8)$$

where E is the energy contained in the pump pulse with a wavelength of λ; $\alpha(\lambda)$ and R_{pump} represent the absorption coefficient and the reflectance of the pump pulse, respectively; A_{eff} is the efficient overlap area of the pump and the probe beam[27,28].

Global fitting of the TA data

As described in the manuscript, the carrier recombination kinetics in the film can be described by the following rate equation:

$$R = -\frac{dN(t)}{dt} = k_3N^3 + k_2N^2 + k_1N \quad (9)$$

where N is the injected carrier density. Therefore, combining equation **7** and **8**, we can obtain the following relation:

$$\frac{dx}{dt} = C^2\varphi^2k_3x^3 + C\varphi k_2x^2 + k_1x \quad (10)$$

Furthermore, based on equation **10**, by globally fitting the transmission change of probe pulse under different excitation intensities, we can extract each order recombination rate constants. To fully avoid the interference of the high-order recombination process, a TRPL decay curve at a low excitation fluence was selected to extract k_1. Notably, since the constant φ is hard to measure, the k_2 and k_3 obtained by this method are actually the lower limits of the real recombination constant.

The relationship between E_b and recombination rate

According to previous reports, the bimolecular electron-hole radiation recombination rate k_2 can be simplified to[29]:

$$k_2 \propto 1 + {a_*}^2 \varepsilon^2 \quad (11)$$

where a_* is the effective Bohr radius, ε is the static dielectric constant of the material. Obviously, the quasi-2D perovskite with high exciton binding energy has a reduced dielectric constant thus show a high k_2.

For Auger recombination, the relationship between E_b and Auger recombination rate are also discussed in previous reports. In a one-dimensional system, it has been proved that k_3 is proportional to the third power of E_b[30].

Moreover, in quasi-2D perovskite materials, due to the enhanced Coulomb interaction, the electron density in the vicinity of a hole is increased while it is decreased for another electron[31]. This nonuniform distribution therefor induce to Coulomb-enhanced Auger recombination. Thus, the k_3 can be written in the form:

$$k_{\mathrm{Auger}} = g_{eeh}\, C_n\, n^2\, p \quad (12)$$

where g_{eeh} is the Coulomb enhancement factor; C_n is the Auger coefficient; n and p are the density of electrons and holes respectively.

References

1. Milot, R. *et al.* Radiative monomolecular recombination boosts amplified spontaneous emission in $HC(NH_2)_2SnI_3$ perovskite films. *J. Phys. Chem. Lett. 7*, 4178-4184 (2016).
2. Cho, H. *et al.* High-efficiency polycrystalline perovskite light-emitting diodes based on mixed cations. *ACS Nano* **12**, 2883-2892 (2018).
3. Jiang, Y. *et al.* Spectra stable blue perovskite light-emitting diodes. *Nat. Commun.* **10**, 1868 (2019).
4. Kumar, S. *et al.* Ultrapure green light-emitting diodes using two-dimensional formamidinium perovskites: Achieving recommendation 2020 color coordinates. *Nano Lett.* **17**, 5277-5284 (2017).
5. Yuan, S. *et al.* Self-assembled high quality CsPbBr3 quantum dot films toward highly efficient light-emitting diodes. *ACS Nano* **12**, 9541-9548 (2018).
6. Wang, H. *et al.* A multi-functional molecular modifier enabling efficient large-area perovskite light-emitting diodes. *Joule* **4**, 1977-1987 (2020).
7. Xie, C., Zhao, X., Ong, E. W. Y. & Tan, Z.-K. Transparent near-infrared perovskite light-emitting diodes. *Nat. Commun.* **11**, 4213 (2020).
8. Zhao, X. *et al.* Large-area near-infrared perovskite light-emitting diodes. *Nat. Photonics* **14**, 215-218 (2020).
9. Chu, S. *et al.* Large-area and efficient perovskite light-emitting diodes via low-temperature blade-coating. *Nat. Commun.* **12**, 147 (2021).
10. Lin, K. *et al.* Perovskite light-emitting diodes with external quantum efficiency exceeding 20 per cent. *Nature* **562**, 245-248 (2018).
11. Wu, T. *et al.* High-performance perovskite light-emitting diode with enhanced operational stability using lithium halide passivation. *Angew. Chem. Int. Ed.* **59**, 4099-4105 (2020).

12. Wang, H. *et al.* Trifluoroacetate induced small-grained CsPbBr3 perovskite films result in efficient and stable light-emitting devices. *Nat. Commun.* **10**, 665 (2019).

13. Zhang, Q. *et al.* Efficient metal halide perovskite light-emitting diodes with significantly improved light extraction on nanophotonic substrates. *Nat. Commun.* **10**, 727 (2019).

14. Zhao, B. *et al.* High-efficiency perovskite–polymer bulk heterostructure light-emitting diodes. *Nat. Photonics* **12**, 783-789 (2018).

15. Xu, W. *et al.* Rational molecular passivation for high-performance perovskite light-emitting diodes. *Nat. Photonics* **13**, 418-424 (2019).

16. Zhao, B. *et al.* Efficient light-emitting diodes from mixed-dimensional perovskites on a fluoride interface. *Nat. Electron.* **3,** 704-710 (2020).

17. Chiba, T. *et al.* Anion-exchange red perovskite quantum dots with ammonium iodine salts for highly efficient light-emitting devices. *Nat. Photonics* **12**, 681-687 (2018).

18. Xu, L. *et al.* A bilateral interfacial passivation strategy promoting efficiency and stability of perovskite quantum dot light-emitting diodes. *Nat. Commun.* **11**, 3902 (2020).

19. Ban, M. *et al.* Solution-processed perovskite light emitting diodes with efficiency exceeding 15% through additive-controlled nanostructure tailoring. *Nat. Commun.* **9**, 3892 (2018).

20. Yang, X. *et al.* Efficient green light-emitting diodes based on quasi-two-dimensional composition and phase engineered perovskite with surface passivation. *Nat. Commun.* **5**, 570 (2018).

21. Shi, D. *et al.* Low trap-state density and long carrier diffusion in organolead trihalide perovskite single crystals. *Science* **347**, 519-522 (2015).

22. Kresse, G. & Furthmuller, J. Efficient iterative schemes for ab initio total-energy calculations using a plane-wave basis set. *Phys. Rev. B: Condens. Matter Mater. Phys*. **54**, 11169-11186 (1996).

23. Monkhorst, H. J. & Pack, J. D. Special points for brillouin-zone integrations. *Phys. Rev. B* **13**, 5188-5192 (1976)

24. Perdew, J. P., Burke,K., Ernzerhof & Generalized M. Gradient approximation made simple. *Phys. Rev. Lett.* **77**, 3865-3868 (1996).

25. Blochl, P. E. Projector augmented-wave method. *Phys. Rev. B*: *Condens. Matter Mater. Phys.* **50**, 17953-17979 (1994).

26. Grimme, S., Antony,J. Ehrlich, S. & Krieg,H. Consistent and accurate ab initio parametrization of density functional dispersion correction (DFT-D) for the 94 elements H-Pu. *J. Chem. Phys.* **132**, 154104 (2010).

27. Rehman, W. *et al.* Charge-carrier dynamics and mobilities in formamidinium lead mixed-halide perovskites. *Adv. Mater.* **27**, 7938-7944 (2015).

28. Chen, Z. *et al.* Recombination Dynamics Study on Nanostructured Perovskite Light-Emitting Devices. *Adv. Mater.* **30**, 1801370 (2018).

29. Lasher, G., & Stern, F. Spontaneous and stimulated recombination radiation in semiconductors. *Phys. Rev.*, **133**, A553-A563 (1964).

30. Wang, F. *et al.* Auger recombination of excitons in one-dimensional systems. *Phys. Rev. B*, **73**, 245424 (2006).

31. Hangleiter, A. & Häcker, R. Enhancement of band-to-band Auger recombination by electron-hole correlations. *Phys. Rev. Lett.*, **65**, 215-218 (1990).
